# Exploring ISAC: Information-Theoretic Insights

**DOI:** 10.3390/e27040378

**Published:** 2025-04-02

**Authors:** Mehrasa Ahmadipour, Michèle Wigger, Shlomo Shamai

**Affiliations:** 1 UMPA, ENS de Lyon, 69342 Lyon, France; 2 LTCI Telecom Paris, IP Paris, 91120 Palaiseau, France; 3 Technion, Haifa 3200003, Israel

**Keywords:** integrated sensing and communication, information theory, radar, capacity–distortion tradeoff, state-dependent channels, multi-user communication, secrecy constraints

## Abstract

This article reviews results from the literature illustrating the bottlenecks and tradeoffs of integrated sensing and communication (ISAC) through the lens of information theory, thus offering a distinct perspective compared to recent works that focus on signal processing, wireless communications, or other related overviews. Different models and scenarios are considered and compared. For example, scenarios where radar sensing is performed at the communication and radar transmitter (mono-static ISAC) and scenarios where the radar receiver differs from the radar transmitter (called bi-static radar). Similarly, we discuss ISAC bottlenecks and tradeoffs both in slowly-varying environments where the main sensing target is described by a single parameter and accordingly, sensing performance is described by detection error probabilities, as well as in fast-varying environments, where the sensing targets are described by vectors and thus vector-valued performance measures such as average distortions like mean-squared errors are used to determine sensing performances. This overview article further also considers limitations and opportunities in network ISAC environments, such as collaborative or interactive sensing, and the influence of secrecy and privacy requirements on ISAC systems, a line of research that has received growing interest over the last few years. For all these scenarios, we provide and discuss precise models and their limitations and provide either bounds or full characterizations of the fundamental information-theoretic performance limits of these systems. Further extensions as well as important open research directions are also discussed.

## 1. Introduction

Integrated sensing and communication (ISAC) represents a transformative paradigm that unifies sensing and communication functionalities into a single system, leveraging shared spectral, hardware, and computational resources. This integration is increasingly crucial in modern technological ecosystems, where efficient resource utilization and enhanced system performance are paramount. ISAC offers notable benefits in applications such as autonomous vehicles, industrial automation, smart cities, and wireless networks.

By reducing latency, improving spectral efficiency, and enhancing situational awareness, ISAC is foundational to emerging technologies like 6G communication and the Internet of Things (IoT). Its dual-purpose design minimizes infrastructure costs while enabling seamless interaction between sensing and communication, paving the way for adaptive and intelligent systems.

As an example of a practical ISAC system, we can consider Wi-Fi technology. Based on the IEEE 802.11 standards, it has delivered significant social and economic benefits. Recently, attention has turned to WLAN sensing—also known as Wi-Fi sensing—which leverages the widespread Wi-Fi infrastructure and ubiquitous signals in our environment to perform various sensing tasks. By employing advanced signal processing techniques, received Wi-Fi signals can be used to detect obstructions, monitor environmental changes, and interpret target movement. Despite these innovations and progress, several challenges remain in current standardization efforts, as evidenced by ongoing work towards IEEE 802.11bf for WLAN sensing and ISAC [1].

In general, designing effective ISAC systems involves achieving simultaneous high-performance sensing and communication. Advanced configurations, such as bi-static radar or multi-terminal ISAC systems, require efficient exchange of sensing information between terminals to enhance collective sensing capabilities rather than relying solely on local data. Furthermore, modern ISAC applications impose additional constraints, such as privacy and security. Balancing these competing requirements necessitates an understanding of the tradeoffs and fundamental performance limits across various system criteria.

Information theory has a rich history and provides a robust framework for analyzing such complex multi-purpose systems and revealing the inherent tensions and tradeoffs in the fundamental limits of the various performances of such systems. Fundamental results from the information theory literature for communication (e.g., data rate, capacity) [2,3,4,5,6,7], detection and hypothesis testing [8,9,10,11,12,13,14,15,16,17,18,19,20], estimation [21,22,23,24], and compression [25,26,27,28,29,30,31] indeed can form the foundation for analyzing ISAC systems. Prior studies have explored tradeoffs between these performance measures in both distributed and non-distributed setups. Some ISAC scenarios, though not explicitly named as such, have been studied within the information-theoretic community under related contexts like “simultaneous data communication and state estimation”. Other scenarios remain unexplored but can benefit from information-theoretic insights derived from analogous setups.

This article aims to synthesize and present both established and emerging information-theoretic results relevant to ISAC systems. Compared to many existing overview ISAC articles that tackle the problem more from a communication and signal processing angle [32,33,34,35,36,37,38,39,40,41], here we focus on information-theoretic results that are more closely aligned with [42,43]. Specifically, we focus on the inherent tradeoffs and fundamental performance limits of ISAC systems, emphasizing coding techniques and proof strategies that enable optimal sensing–communication tradeoffs. We would like to point out that the information-theoretic models studied in this survey are very general, and can be specialized to different practical scenarios of interest. Keeping the model and results general allows us to derive broadly based conclusions and also cover a larger range of application scenarios.

Specifically, in this article, we start with a a brief historical perspective of ISAC (Section 2), followed by a first technical section (Section 3) that considers a canonical ISAC point-to-point setup with a single Tx wishing to communicate to a single Rx and where the sensing task is to estimate a state sequence (such as the accelerations of an obstacle) up to a desired distortion. We will start by discussing results on simple memoryless channel models, and then move on to very general models with memory. All the results discussed in this Section 3 illustrate the inherent tradeoff between the sensing and communication tasks encountered in such ISAC systems, while Section 3 considers a mono-static radar setup, i.e., the sensing task is performed at the Tx of the communication system, the subsequent section, Section 4, considers bi-static radar models where sensing is performed at the Rx. Section 5 further generalizes the setup to multiple Txs or Rxs and sensing at multiple terminals. Not only is the communication problem of formidable difficulty in these setups but the sensing task is also significantly more involved, as now collaborative and interactive sensing strategies can be applied to provide remote terminals with sensing information gathered at other terminals. As we shall see, in such scenarios, it does not just suffice to exchange sensing information using standard communication schemes, but instead the code construction previously only used for data communication now needs to be adapted to also enable the collaborative sensing tasks. Moreover, given the distributed sensing information that has to be conveyed from certain terminals to others, network joint source-channel coding schemes become essential to attain good sensing performances. The subsequent Section 6, then describes how the above ISAC schemes and performance limits need to be adapted so as to ensure secrecy of only messages or of states and sensing targets/states. The last technical section (Section 7) of this overview then takes a different approach to the sensing task, assuming that the sensing task consists of detecting a single parameter (and not estimating a state vector as in the previous chapters) which determines the behavior of the sensing target. This problem seems to be slightly more challenging but first instructive results are presented, in particular, when the Tx is restricted to non-adaptive coding schemes where the backscattered signals can be used for the sensing task but not to produce the subsequent inputs. The overview article is then concluded with concluding remarks.

There exists a large body of studies on other aspects of ISAC systems, for example, the works [44,45,46,47,48,49,50,51,52,53,54,55] have studied ISAC from a more communication-theoretic perspective in environments where Txs and Rxs are equipped with multiple antennas, in particular, in so-called massive multi-input and multi-output (MIMO) systems. Interesting research directions result in these MIMO systems regarding whether the smart selection of beamformers allows a reduction in the tradeoff between communication and sensing performances and how the tradeoff is influenced by the choice of the antenna distance. Recent initiatives to improve the understanding of ISAC systems of course also include learning-based studies. The tutorial in [41] provides a comprehensive overview of works using deep-learning based techniques and the summary of the reviewed results are provided in Table 1.

### Notation

In this survey, we shall use class notation for our mathematical expressions. For example, upper-case letters like *X* denote random quantities, while lower-case letters like *x* represent their deterministic realizations. Sets are represented using calligraphic font (e.g., X). The *n*-tuples $\left(X_{1},\ldots ,X_{n}\right)$ and $\left(x_{1},\ldots ,x_{n}\right)$ are abbreviated as $X^{n}$ and $x^{n}$, respectively. Similarly, the n−t-tuples $\left(X_{t+1},\ldots ,X_{n}\right)$ and $\left(x_{t+1},\ldots ,x_{n}\right)$ are written as $X_{t+1}^{n}$ and $x_{t+1}^{n}$. *Independent and identically distributed* is abbreviated as *i.i.d.*, and *probability mass function* as *pmf*. The conditional probability is written as $P_{XY|UV}\left(x,y|u,v\right)$, and $P_{X}\left(\cdot \right)$ represents the pmf of a finite random variable *X*. The expectation of a random variable *X* is denoted by E[X]. R and $R_{0}^{+}$ denote the sets of real numbers and non-negative real numbers, respectively. log typically represents the base-2 logarithm, accordingly information measures are measured in terms of *nats*. The operator ⊕ typically indicates XOR (binary addition modulo 2).

The argmin represents the set of minimizers of a function. $\bar{\lim}$ and $\underline{\lim}$ denote the limit superior and limit inferior as n→∞. The operator $f^{\left(n\right)}$ represents a transformation or operation on *n*-letter sequences. The term esssup refers to the essential supremum in measure theory. Entropy, conditional entropy, and mutual information are denoted by H(·), H(·|·), and I(·;·), respectively. When the probability mass function (pmf) is not clear from the context, it is included as a subscript, e.g., $H_{P}\left(\cdot \right)$. The Kullback–Leibler divergence between two pmfs is denoted by D(·∥·).

## 2. Pre-ISAC: Sensing (Radar) vs. Communication

### 2.1. Radar Systems

Radar is a system that utilizes radio waves to learn about positions, motions, or the mere presence of target objects in an environment through the analysis of backscattered signals. In fact, a radar terminal radiates a waveform that propagates through space until it reaches a target, where it is reflected in a way that depends on the properties of the target. The radar terminal collects and analyzes the backscattered signals so as to gain information about these properties. In the radar system, if the presence and position of a target are already known, the transmitter tries to steer all the energy of the transmitted waveform towards the target, so as to obtain more information through the backscattered waveform. Radar thus uses line-of-sight (LoS) techniques. Traditional radar systems mainly operate within the 24–79 GHz frequency band.

Sensing tasks can be roughly classified into three categories, detection, estimation, and recognition, which are all based on collecting signals/data concerning the sensed objects. Detection refers to making decisions on an object’s *state* given some observations, such as the presence/absence of the target or other events related to the target. The detection problem can be modeled as a binary or multi-hypothesis testing problem. In the binary hypothesis testing problem, as an example, one selects from two hypotheses: the alternative hypothesis $H_{1}$ and the null hypothesis $H_{0}$. Detection metrics are the probability that $H_{1}$ holds but the detector chooses $H_{0}$ (often denoted miss-detection probability), and the probability that $H_{0}$ holds but the detector chooses $H_{1}$ (often denoted false-alarm probability).

Estimation refers to extracting valuable parameters, typically with continuous alphabets, of the sensed object from observations. For example, distance/velocity/angle/quantity/ size of targets are possible parameters that a radar system desires to estimate. Various interesting performance metrics exist for estimation, whose suitability depends on the application. Prominent examples are the mean squared error (MSE) metric, which measures the expected squared-error of the estimated parameter to the ground truth parameter. Note that in the case of *unbiased estimators*, i.e., estimators $\hat{S}$ whose conditional expectation is always equal to the true parameter *S*, $E[\hat{S}|S]=S$, the Cramér–Rao bound (CRB) expresses an interesting lower bound on the MSE that can be attained by any unbiased estimator.

### 2.2. Wireless Communication Systems

In a communication system, a transmitter (Tx) aims to transfer either data bits or source samples (such as audio or video file samples) to a distant receiver (Rx). The data or source information is encoded onto a transmitted waveform, which the receiver then collects and analyzes to estimate the transmitted information. Performance metrics commonly considered for communication systems include energy or spectral efficiency, which measure how many bits of information are communicated using a given energy budget or bandwidth, respectively.

For data transmission, robustness of communication is typically measured by the bit-error rate (BER), symbol-error rate (SER), or frame-error rate (FER), which indicate the likelihood of errors in the received data due to channel disturbances. In source communication, robustness is either measured by the bit-error rate or more often by distortion metrics such as the average mean-squared error. These performance metrics are especially pertinent in traditional wireless communication systems, which predominantly operate in the 2.4 GHz band. The main differences between radar and communication systems are shown in Table 2.

### 2.3. Coexisting Communication and Radar Systems

Early approaches [76] modulated the communication bits on a missile range radar pulse interval. Interference rejection and robustness in multipath fading environments, inherent properties of spread spectrum systems, also made chirp signaling (used in the radar application) very active in the expanding wireless communications market.

Another approach in [77] proposed as early as 1962 is based on chirp signals, which were originally proposed for both analog and digital communication [78] but are also commonly used in radar applications. These works can be categorized as the first steps towards *untegrated sensing and communication* (*ISAC*).

Since then, significant evolution has lead to an entire set of pre-ISAC systems, see [32] where a category of solutions is revisited. Some straightforward solutions are called *non-overlapped resource allocation*. In subsequent information-theoretic models, as we will see later, such a system corresponds to time- or resource-sharing between communication and sensing; we shall call this *basic time-sharing* (*TS*), and with a minor modification, we will introduce *improved time-sharing*.

A common but naive approach to address sensing and communication is to separate the two tasks into independent systems and split the available resources, such as bandwidth and power, between them so that they do not interfere.

Time-division ISAC can be conveniently implemented into existing commercial systems by splitting the transmission duration into radar and radio cycles, for example [79]. For radar sensing, frequency-modulated continuous waveform (FMCW) with up-and-down-chirp modulations is used, while various different modulation schemes (e.g., BPSK, PPM) can be used for communication.

In an orthogonal frequency division multiplexing (OFDM) system, frequency-division ISAC can be implemented by allocating different communication and sensing tasks to specific subcarriers, depending on the channel conditions and power budget of the Tx [80].

Similarly, the 3GPP/5G-NR standards were originally designed primarily for communication but have evolved to accommodate additional functionalities, such as positioning and sensing. The 5G-NR standard primarily uses OFDM due to its flexibility and efficiency, and its inherent structure can also be exploited for sensing tasks such as radar-like functions and localization. In practice, enhancements (e.g., pilot designs and advanced signal processing techniques) are introduced to extract sensing information from these communication signals. Meanwhile, orthogonal time frequency space (OTFS) modulation [81,82] has attracted increasing interest as an alternative to OFDM, placing symbols in the delay–Doppler domain to handle high-mobility channels more robustly. By directly leveraging delay and Doppler features, OTFS can inherently support sensing-like operations, making it appealing for ISAC in future releases of 5G and beyond. In information-theoretic studies, these models are often incorporated into resource-splitting approaches. The ideal goal of ISAC is to further serve both tasks, as discussed in this work. For details on the evolution of 3GPP, see [83].

**Figure 1:**
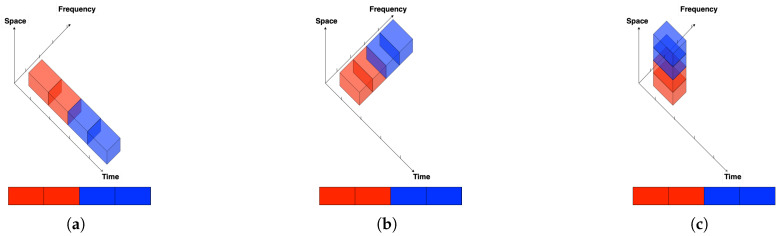
The red cubes demonstrate the communication waveform and the blue cubes demonstrate the sensing waveform. (**a**) Time-sharing. (**b**) Frequency-sharing. (**c**) Spatial-sharing.

ISAC with non-overlapped resources can also be implemented over orthogonal spatial resources, e.g., different antenna groups [84]. Thus, non-overlapping resource allocation can be performed in the time, frequency, or spatial domains, as illustrated also in Figure 1.

From an information-theoretic perspective, we examine pre-ISAC and non-overlapping schemes through two baseline approaches: the basic time-sharing (TS) scheme and the improved time-sharing (TS) scheme. The basic TS scheme represents the non-overlapping resource allocation strategy, which divides its resources (time, bandwidth, or spatial dimensions) between the following two modes:

- *Sensing mode*
  The system aims to design a suitable waveform to attain the minimum possible distortion. In this model, the waveform is translated into an input distribution; thus, the input probability mass function (pmf) $P_{X}$ is chosen to minimize the distortion, and hence, the minimum distortion is achieved. The communication rate is zero.
- *Communication mode*
  The system is designed to transfer as much reliable data as possible. Therefore, the input distribution is chosen to maximize the rate and communicates rate equals channel capacity. The estimator is set to a constant value regardless of the feedback and the input signals. The mode thus suffers from a large distortion.

The improved TS scheme still performs a sort of non-overlapped resource allocation, but resources are not exclusively dedicated to only sensing or only communication. It is simply that one of these tasks is prioritized. The second baseline scheme is improved TS scheme and can simultaneously perform the communication and sensing tasks. This scheme time-shares between the following modes.

- *Sensing mode with communication* The input pmf $P_{X}$ is chosen to achieve minimum distortion. At the same time, the transmitter is also equipped with a communication encoder. It uses this input pmf to simultaneously transmit data at the rate given by the input-output mutual information of the system.
- *Communication mode with sensing* The input distribution is chosen to maximize the communication rate, i.e., achieve the capacity of the channel. The transmitter is, however, also equipped with a radar estimation device that optimally guesses the state sequence based on the transmitted and backscattered signals.

### 2.4. Integrated Sensing and Communication (ISAC)

The ISAC concept originated from observations in communication systems where backscattered signals were typically ignored and not utilized. Subsequent studies revealed that these backscattered signals, though initially overlooked, could provide valuable information that can help the transmitter(s) (Tx(s)) to improve communication performance or simplify coding schemes. In fact, backscattered signals can help any transmitting terminal to better estimate current and future channel conditions at the intended receivers (Rxs) or to identify the receivers’ uncertainty about the transmitted data. Accordingly, they can improve transmission performance by adapting future transmissions to the uncertainties to resolve or future channel conditions. Such strategies allow a decrease in error probabilities and simplify coding schemes, and when channels vary only slowly in time, with high dependencies between channel conditions at different time, adaptive schemes can even achieve higher reliable rates, i.e., improve capacity.

It is not hard to see that in typical ISAC scenarios, all non-extreme operating points of the basic and improved TS schemes are highly suboptimal compared to optimal integrated schemes.

## 3. Mono-Static ISAC with Sensing Distortion

In this section, we introduce a first simple information-theoretic model of ISAC that allows the obtaining of a convenient expression for the information-theoretic limits and tradeoffs for ISAC point-to-point channels with a single Tx and a single Rx. Sensing performance is measured by an arbitrary distortion function, as is typically used by information theorists in rate–distortion theory to formalize lossy compression systems or joint source-channel coding. The model is powerful in the sense that it allows the inclusion of desired properties both from a sensing and a communication perspective. For example, any arbitrary number of sensing targets can be modeled, as well as a wide range of sensing metrics. Similarly, depending on the specific situation one wishes to analyze, this model can describe arbitrary communication and radar channels, which may or may not depend on the sensing targets. Moreover, the model allows the inclusion of arbitrary (perfect or imperfect) channel state information (CSI) at the Rx and arbitrary instantaneous but causal CSI at the Tx, which typically are obtained by transmission of independent pilot signals. However, it should be noted that the proposed model can only model channels and targets that evolve in a memoryless fashion. Moreover, certain sensing performances like detection probabilities cannot be described using distortion conditions. To remedy this latter drawback, in Section 7, we introduce a related problem where sensing performance is measured in terms of detection error probabilities.

It should be noted that a very similar model was also considered in [53] for a multi-antenna Gaussian fading channel. In this related work, sensing performance is, however, not measured in terms of distortion but by an averaged inverse Fisher information, which is motivated by the well-known Cramér–Rao bound. In particular, in [53], I-MMSE is introduced as a unifying relation between distortion-based sensing (MMSE) and communications (mutual information). It seems more difficult to determine the fundamental limits of this basic ISAC model under this related sensing criterion; however, the authors of [53] were able to determine some extreme points of this tradeoff: the points of optimum communication performance or optimum sensing performance.

### 3.1. The Memoryless Model

**Figure 2:**
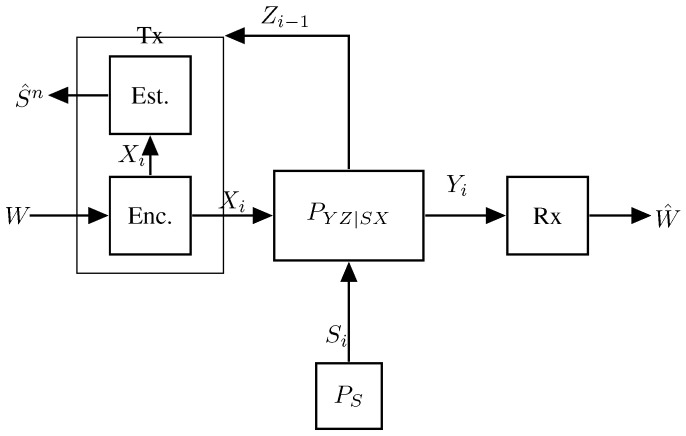
The first information-theoretic ISAC model.

The first information-theoretic model for a single-Tx single-Rx ISAC system was introduced by Kobayashi, Caire, and Kramer in [56] and is depicted in Figure 2.

In this model, a single Tx wishes to communicate a message *W* of nR independent and uniform data bits to the single Rx by communicating over *n* uses of a state-dependent discrete memoryless channel. The state sequence $S_{1},\ldots ,S_{n}$ models the parameters one wishes to estimate (e.g., the accelerations of a given target) and in this model, are assumed i.i.d. according to a given and known distribution $P_{S}$. At the same time, the Tx also aims to estimate the state sequence $S_{1}^{n}=\left(S_{1},\ldots ,S_{n}\right)$ from generalized feedback signals, which here model the backscatterers observed at the Tx. The communication channel and the radar channel (i.e., the generation of the backscatterers) are jointly modeled by a DMSC with a stationary channel law $P_{YZ|XS}\left(y,z|x,s\right)$. That means, if at a given time i∈{1,…,n} (more precisely for a given channel use *i*), the Tx feeds input $X_{i}=x_{i}$ to the channel and the state realization is $S_{i}=s_{i}$, then the Rx’s time-*i* observed channel output $Y_{i}$ and the generalized feedback signal $Z_{i}$ backscattered to the Tx are generated according to the conditional pmf $P_{YZ|XS}\left(\cdot ,\cdot |x_{i},s_{i}\right)$, irrespective of the past inputs, outputs, and state realizations.

The transmitter produces its (potentially random) channel inputs $X_{1},\ldots ,X_{n}$ as a function of the message *W* and the backscattered signals. So, it produces the time-*i* input as $X_{i}=\phi_{i}\left(W,Z_{1},\ldots ,Z_{i-1}\right)$, for i=1,…,n. The receiver observes the channel outputs $Y_{1},\ldots ,Y_{n}$ corresponding to these inputs and based on the entire sequence produces a guess of the message $\hat{W}=g\left(Y_{1},\ldots ,Y_{n}\right)$.

Based on the backscattered sequence $Z_{1},\ldots ,Z_{n}$ and its produced inputs $X_{1},\ldots ,X_{n}$, the transmitter also produces the state estimates $\left({\hat{S}}_{1},\ldots ,{\hat{S}}_{n}\right)=h\left(X_{1},\ldots ,X_{n},Z_{1},\ldots ,Z_{n}\right)$. The quality of these state estimates is measured by the expected average per-block distortion

(1) $$\Delta^{\left(n\right)}:=E\left[d\left(S^{n},{\hat{S}}^{n}\right)\right]=\frac{1}{n}\sum_{i=1}^{n}E\left[d\left(S_{i},{\hat{S}}_{i}\right)\right]$$

where $d:S\times \hat{S}\mapsto R_{0}^{+}$ is a given bounded *distortion function*:

(2) $$\max_{\left(s,\hat{s}\right)\in S\times \hat{S}}d\left(s,\hat{s}\right)<\infty .$$

Examples of commonly used distortion functions are the Hamming distortion $d\left(s,\hat{s}\right)=1\left\{s\neq \hat{s}\right\}$, which measures the fraction of wrongly reconstructed symbols, or the mean-squared error distortion $d\left(s,\hat{s}\right)={\left(s-\hat{s}\right)}^{2}$, which measures the average squared distance between the reconstruction and source sequences.

A last feature of this model is a cost constraint (such as a power constraint) on the input sequence, as imposed by many practical communication systems. These cost constraints can often be expressed as

(3) $$E\left[b\left(X^{n}\right)\right]=\frac{1}{n}\sum_{i=1}^{n}E\left[b\left(X_{i}\right)\right]$$

for some given cost functions $b:X\mapsto R_{0}^{+}$. In the case of an input power constraint, the cost function $b\left(x\right)=x^{2}$ is implied for radio channels where the power is proportional to the square of the emitted signal (which represents the electromagnetic field).

### 3.2. The Capacity–Distortion–Cost Tradeoff

The goal of information-theoretic studies is to identify the optimal performance that can be achieved by choosing the best system implementation under given modeling assumptions. In the present case, we consider the memoryless model introduced in the previous section, Section 3.1, and wish to determine the performance of the optimal data encoders and decoders, and state estimators. We will limit the study to encoders/decoders that have arbitrarily small error probabilities when the blocklength *n* grows without bounds.

**Definition** **1.** *A rate–distortion–cost tuple (R,D,B) is said achievable if there exists a sequence (in n) of encoding, decoding, and estimation functions $\left(\phi_{1},\ldots ,\phi_{n},g,h\right)$ that simultaneously satisfy*

(4a) $$\lim_{n\to \infty }\mathrm{Pr}\left[\hat{W}\neq W\right]=0,$$

(4b) $$\underset{n\to \infty }{\bar{\lim}}\Delta^{\left(n\right)}\leq D,$$

(4c) $$\underset{n\to \infty }{\bar{\lim}}\frac{1}{n}\sum_{i=1}^{n}E\left[b\left(X_{i}\right)\right]\leq B.$$

*The* capacity–distortion–cost tradeoff C(D,B) *is the largest rate R such that the rate–distortion–cost triple (R,D,B) is achievable.*

The main result of Kobayashi et al. in [56], see Theorem 1 below, provides an exact characterization of C(D,B). It assumes that the receiver has perfect state information at the receiver. Here, we give the more general result that also includes the case without receiver state information. Note that this model is more general because any kind (even imperfect) of receiver-side information can be provided to the receiver as part of the output *Y*, in which case the original results of [56] are recovered. For a more detailed discussions on this, see [63].

A first step to obtain this result is to describe the optimal estimator, which in the present memoryless model is pleasingly simple, because it operates on a symbol-by-symbol basis. That means, estimate ${\hat{S}}_{i}$ of the *i*-th state symbol $S_{i}$ is solely based on the *i*-th input $X_{i}$ and feedback signal $Z_{i}$.

**Lemma** **1.** [56] *Define the function*

(5) $$\begin{array}{c} {\hat{s}}^{*}\left(x,z\right):=\arg\min_{s^{\prime }\in \hat{S}}\sum_{s\in S}P_{S|XZ}\left(s|x,z\right)d\left(s,s^{\prime }\right), \end{array}$$

*where ties can be broken arbitrarily, and*

(6) $$P_{S|XZ}\left(s|x,z\right)=\frac{P_{S}\left(s\right)P_{Z|SX}\left(z|s,x\right)}{\sum_{\tilde{s}\in S}P_{S}\left(\tilde{s}\right)P_{Z|SX}\left(z|\tilde{s},x\right)}.$$

*Irrespective of the choice of encoding and decoding functions, distortion *$\Delta^{\left(n\right)}$ *in* (4b) *is minimized by the estimator*

(7) $$h^{*}\left(x^{n},z^{n}\right):=\left({\hat{s}}^{*}\left(x_{1},z_{1}\right),{\hat{s}}^{*}\left(x_{2},z_{2}\right),\ldots ,{\hat{s}}^{*}\left(x_{n},z_{n}\right)\right).$$

Note that the function ${\hat{s}}^{*}\left(\cdot ,\cdot \right)$ only depends on the SDMC channel law $P_{YZ|SX}$ and the state distribution $P_{S}$.

To be used later, define the following sets of input distributions:

(8a) $$\begin{array}{cc} P_{B} & =\left\{P_{X}\;|\sum_{x\in X}P_{X}\left(x\right)b\left(x\right)\leq B\right\}, \end{array}$$

(8b) $$\begin{array}{cc} P_{D} & =\left\{P_{X}\;|\sum_{x\in X}P_{X}\left(x\right)E\left[d(S,{\hat{s}}^{*}\left(X,Z\right))|X=x\right]\leq D\right\}. \end{array}$$

Then, the minimum distortion for a given cost B is given by

(9) $$D_{\min}\left(B\right):=\min_{P_{X}\in P_{B}}\sum_{x\in X}P_{X}\left(x\right)E\left[d(S,{\hat{s}}^{*}\left(X,Z\right))|X=x\right].$$

The main result in [56,63] is the following theorem:

**Theorem** **1.** *The capacity–distortion–cost tradeoff of an SDMC $P_{YZ|SX}$ with state distribution $P_{S}$ is:*

(10) $$C\left(D,B\right)=\max_{P_{X}\in \left(P_{B}\cap P_{D}\right)}I\left(X;Y\right),\;D\geq D_{\min}\;\;B\geq 0.$$

**Remark** **1.** *Note that the above result also remains valid if the expected distortion constraint* (4b) *is replaced by an excess distortion constraint where the probability that the sequences exceed average distortion* D *is required to vanish asymptotically in the blocklength n. The work in [57] considered such an excess distortion criterion but imposed weaker constraints where both the excess distortion probability and the decoding error probability do not necessarily need to vanish asymptotically but can simply be bounded by given positive constants* δ,ϵ∈(0,1). *The analysis in [57] showed that the fundamental limits remain unchanged when the sum of both allowed error probabilities δ+ϵ<1. If maximum error probabilities are considered instead of average error probabilities (over messages), then the results remain for all ϵ,δ∈(0,1), and the so-called strong converse holds. Note that similar non-zero error probability behaviors apply also to only communication but no sensing, because communication takes place over a compound channel, see [20].*

It has been shown in [64] that the rate–distortion tradeoff function C(D,B) is non-decreasing and concave in $D\geq D_{\min}$ and B≥0, and for any B≥0 saturates at the channel capacity without distortion constraints $C_{\mathrm{NoDist}}\left(B\right)$. For many channels, given B≥0, the tradeoff C(D,B) is strictly increasing in D until it reaches $C_{\mathrm{NoDist}}\left(B\right)$. However, for SDMBCs and costs B≥0 where the capacity-achieving input distribution $P_{X_{\max}}:={\mathrm{argmax}}_{P_{X}\in P_{B}}I\left(X;Y|S\right)$ also achieves minimum distortion $D_{\min}\left(B\right)$ in (9), the capacity–distortion tradeoff is constant, irrespective of the allowed distortion D. This is in particular the case when the expected distortion $Ed(S,{\hat{s}}^{*}\left(X,Z\right))$ does not depend on the input distribution $P_{X}$.

To understand the result in Theorem 1, consider the example of a real Gaussian channel with Rayleigh fading and noisy feedback. (For more examples, see [56,64].) The channel output is thus given by:

(11) $$Y_{i}=S_{i}X_{i}+N_{i},$$

where $X_{i}$ is the channel input satisfying ${\bar{\lim}}_{n\to \infty }\frac{1}{n}\sum_{i}E|X_{i}{|}^{2}\leq B=10$ dB, and both sequences $\left\{N_{i}\right\}$ and $\left\{S_{i}\right\}$ are independent of each other and i.i.d. Gaussian with zero mean and unit variance. The Tx observes the noisy feedback

(12) $$Z_{i}=Y_{i}+N_{fb,i},$$

where $\left\{N_{fb,i}\right\}$ are i.i.d. zero-mean Gaussian of variance $\sigma_{\mathrm{fb}}^{2}\geq 0$. We consider the quadratic distortion measure $d\left(s,\hat{s}\right)={\left(s-\hat{s}\right)}^{2}$.

The capacity of this channel is achieved with a Gaussian input $X_{\max}∼N\left(0,B\right)$, and thus the communication mode with sensing achieves the rate–distortion pair

(13) $$C_{\mathrm{NoEst}}\left(B\right)=\frac{1}{2}E\ln(1+|S{|}^{2}B)=1.213,\;$$

(14) $$D_{\max}\left(B\right)=E\frac{\left(1+\sigma_{fb}^{2}\right)}{1+|X_{\max}{|}^{2}+\sigma_{fb}^{2}}=0.367,$$

where the numerical values correspond to $\sigma_{\mathrm{fb}}^{2}=1$ and P=10 dB and the logarithm here is with respect to the natural unit and thus, measured in *nats*.

Minimum distortion $D_{\min}$ is achieved by 2-ary pulse amplitude modulation (PAM), and thus, the sensing mode with communication achieves the rate–distortion pair $(R_{\min}\left(B\right),$$D_{\min}\left(B\right))=\left(0.733,\frac{1+\sigma_{\mathrm{fb}}^{2}}{1+P+\sigma_{\mathrm{fb}}^{2}}=0.166\right)$ where the numerical value again corresponds to $\sigma_{\mathrm{fb}}=1$ and B=10 dB. Next, they characterize the performance of the basic TS baseline scheme. The best constant estimator for this channel is $\hat{s}=0$, and the communication mode without sensing achieves the rate–distortion pair $\left(C_{\mathrm{NoDist}}\left(B\right),D_{\mathrm{trivial}}\left(B\right)=1\right)$. The sensing mode without communication achieves the rate–distortion pair $\left(0,D_{\min}\left(B\right)\right)$.

**Figure 3:**
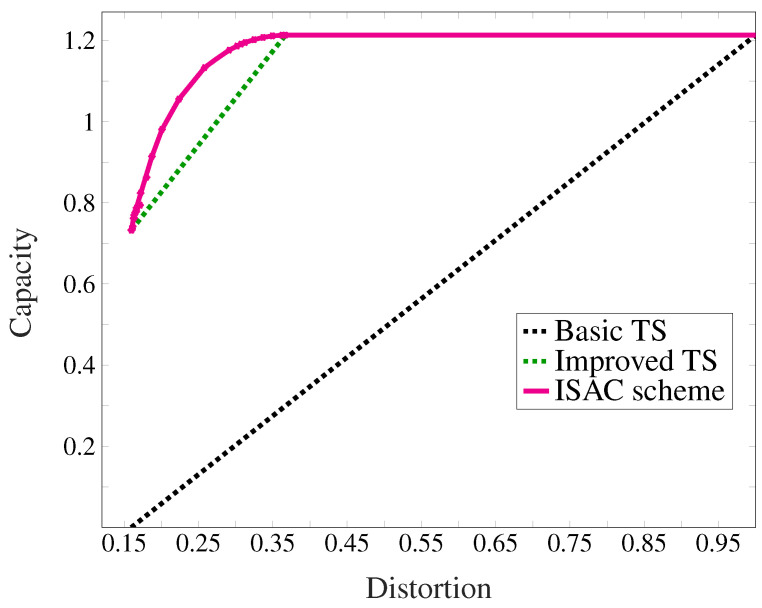
Capacity–distortion tradeoff of fading AWGN channel with B=10 dB and $\sigma_{fb}^{2}=1$ (Rate is measured in *nats*).

In Figure 3, the rate–distortion tradeoff achieved by these two TS baseline schemes is compared with a numerical approximation of the capacity–distortion–cost tradeoff C(D,B) of this channel. As previously explained, C(D,B) also passes through the two end points $\left(R_{\min}\left(B\right),D_{\min}\left(B\right)\right)$ and $\left(C_{\mathrm{NoEst}}\left(B\right),D_{\max}\left(B\right)\right)$ of the improved TS scheme. To obtain a numerical approximation of the points on C(D,B) in between these two operating points an alternating optimization method similar to the Blahut–Arimoto algorithm is used in [56].

### 3.3. Log-Loss Distortion

The work in [58] considered a related setup, where distortion is measured in terms of log-loss distortion. The goal of sensing is thus rather to obtain a soft estimate, i.e., probability distribution $Q_{{\hat{S}}^{n}|X^{n}Z^{n}}\left(\cdot |x^{n},z^{n}\right)$ for the state estimate, instead of a state sequence ${\hat{S}}^{n}$. The model described in the previous section can be adapted to account for a log-loss distortion constraint simply, where in particular the achievability criterion (4b) has to be replaced by the following requirement:

(15) $$\underset{n\to \infty }{\bar{\lim}}\frac{1}{n}\sum_{i=1}^{n}E\left[\log\frac{1}{Q_{{\hat{S}}^{n}|X^{n}Z^{n}}\left(S^{n}|X^{n},Z^{n}\right)}\right]\leq D.$$

In analogy to Lemma 1, it can be argued that the optimal log-loss estimator $Q_{{\hat{S}}^{n}|X^{n}Z^{n}}$ is in product form $Q_{\hat{S}|XZ}^{⊗n}$ and given by the posterior probability in (6)

(16) $$Q_{\hat{S}|XZ}^{*}\left(s|x,z\right)=P_{S|XZ}\left(s|x,z\right)=\frac{P_{S}\left(s\right)P_{Z|SX}\left(z|s,x\right)}{\sum_{\tilde{s}\in S}P_{S}\left(\tilde{s}\right)P_{Z|SX}\left(z|\tilde{s},x\right)},$$

which is solely determined by the channel law and the state distribution but not by the utilized coding scheme.

We can thus conclude that the equivalent of the capacity–distortion–cost tradeoff for log-loss distortion is:

**Theorem** **2.** 
(17) $$C_{\mathrm{LogLoss}}\left(D,B\right)=\max_{\begin{array}{c} P_{X}\in P_{B}:\\ H(S|XZ)\leq D \end{array}}I\left(X;Y\right),$$

*where the entropy and the mutual information are calculated according to the joint law $P_{SXYZ}=P_{S}P_{X}P_{YZ|XS}$.*

Note once more that any kind of receiver-side information can be incorporated in the received signal *Y*, and thus can be treated within the exposed framework. Note further that [58] considers a slightly different approach where the distortion constraint needs to be satisfied for any realization of the message W=w. Interestingly, the two models lead to the same capacity–distortion–cost tradeoff and under both models, the optimal estimator is the posterior estimator.

### 3.4. Finite Blocklength Results

A similar model as introduced in [56] is considered in [59]. Definition 1 needs to be adapted for the nonasymptotic regime as follows:

**Definition** **2.** *Given a blocklength n, the rate–distortion–error triple (R,D,ϵ) is said to be achievable if there exist encoding, decoding, and estimation functions $\left\{f^{\left(n\right)},g^{\left(n\right)},h^{\left(n\right)}\right\}$ satisfying*

(18) $$\begin{array}{cc} \frac{1}{n}\log_{2}\left(W\right) & \geq R, \end{array}$$

(19) $$\begin{array}{cc} \epsilon^{\left(n\right)} & \leq \epsilon , \end{array}$$

(20) $$\begin{array}{cc} \Delta^{\left(n\right)} & \leq D. \end{array}$$

The main results of [59] include the derivation of achievability and converse bounds on the rate–distortion–error tradeoff in the finite blocklength regime.

**Theorem** **3.** *Given a blocklength n, the rate–distortion–error tradeoff (R,D,ϵ) is achievable if there exists a $P_{X}$ and a constant K>0 such that the following conditions are satisfied:*

(21) $$\begin{array}{cc} R & \leq I\left(X;Y\right)-\sqrt{\frac{V}{n}}Q^{-1}\left(\epsilon -\beta_{u}\right)-\frac{K\log(n)}{n}, \end{array}$$

(22) $$\begin{array}{cc} D & \geq \sum_{x\in X}\sum_{s\in S}\sum_{z\in Z}d\left(s,{\hat{s}}^{*}\left(x,z\right)\right)P_{X}\left(x\right)P_{S}\left(s\right)P_{Z|XS}\left(z|x,s\right), \end{array}$$

*where*

$$\beta_{u}:=\frac{1}{nK}+\frac{0.7975T}{\sqrt{nV^{3}}},$$

*and the mutual information I(X;Y) and the central moments V and T are defined based on the joint pmf $P_{XY}\left(x,y\right)=P_{X}\left(x\right)P_{Y|X}\left(y|x\right)$. Conversely, any rate–distortion–error triple (R,D,ϵ) is not achievable if for all δ>0 and pmfs $P_{X}$ satisfying the distortion condition, the following lower bound holds:*

$$R\geq I\left(X;Y\right)-\sqrt{\frac{V}{n}}Q^{-1}\left(\epsilon +\beta_{l}\right)+\frac{\log(n)}{2n}-\frac{\log\delta }{n},$$

*where*

$$\beta_{l}:=\frac{0.7975T}{\sqrt{nV^{3}}}+\frac{\delta }{\sqrt{n}}.$$

Note that here, distortion is measured as an expected distortion over all messages. For a small number of messages, i.e., small blocklengths *n*, the encoder and decoder might need additional randomness to construct the desired distribution. A different approach was taken in [85], where sensing performance is measured with an excess distortion criteria.

**Example** **1.** *Consider a binary channel with a multiplicative Bernoulli state:*

(23) Y=SX,

*where all alphabets are binary X=S=Y∈{0,1}, the state is Bernoulli-q with q∈(0,1) and the feedback is perfect, i.e., Z=Y. We consider the Hamming distortion measure $d\left(s,\hat{s}\right)=s⊕\hat{s}$.*

**Figure 4:**
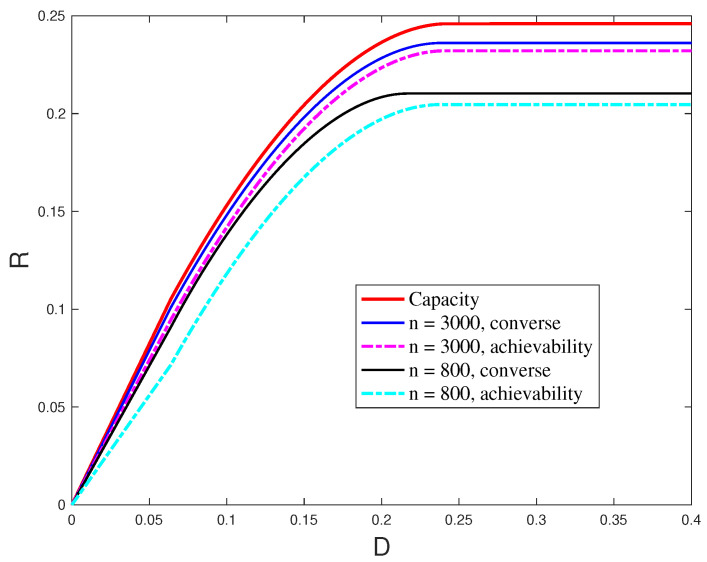
Achievability and converse bounds on the rate–distortion–error trade-off for $\epsilon =10^{-3}$, q=0.4, K=0.5 and different values of the blocklength *n*.

*Figure 4 illustrates the achievability and converse bounds in the above theorem for $\epsilon =10^{-3}$, q=0.4, K=0.5. As can be seen from this figure, the bounds are tight for large values of n. Note that for q=0.4, the capacity of the channel is C=0.246 and the achieved distortion is $D_{comm}=0.2432$.*

### 3.5. Channels with Memory

The previous sections assumed a memoryless stationary model both for the channel and the distribution of the target/state that the transmitter wishes to estimate. The work [60] relaxed both assumptions and considered a general model, where the state process $\left\{S_{t}\right\}$ follows an arbitrary joint distribution and the channel is characterized by a general sequence of transition laws $P_{Y_{i}Z_{i}|X^{i}S^{i}Z^{i-1}Y^{i-1}}$, for i=1,2,…. The distortion constraint is also generalized beyond average block distortion constraints by requiring that

(24) $$p-\underset{n\to \infty }{\bar{\lim}}\frac{1}{n}d\left(S^{n},{\hat{S}}^{n}\right)\leq D,$$

for a general non-negative distortion function d(·,·).

Using Han and Verdu’s information spectrum method, [60] characterized the capacity–distortion tradeoff (Additional cost constraints can be included in the model and the results in a standard way) for this setup with memory. It is given by [60]

(25) $$C\left(D\right):=\underset{{\left\{P_{X^{n}}\right\}}_{n}}{\sup}p-\underset{n\to \infty }{\underline{\lim}}\frac{1}{n}i\left(X^{n};Y^{n}\right)$$

where the supremum is over all input distributions $\left\{P_{X^{n}}\right\}$ and estimators $\left\{{\hat{S}}^{n}\left(Z^{n},X^{n}\right)\right\}$ satisfying ${\bar{\lim}}_{n\to \infty }\frac{1}{n}d\left(S^{n},{\hat{S}}^{n}\left(X^{n},Z^{n}\right)\right)\leq D$. Here, $i(X^{n};Y^{n})$ is the previously defined information density between sequences $X^{n}$ and $Y^{n}$.

A slightly different model was considered in [86], where the transmitter has to estimate the state sequence in an online manner, i.e., a state estimate ${\hat{S}}_{i}$ has to be produced after having produced the time-i channel input $X_{i}$ and having observed the time-i feedback signal $Z_{i}$. The capacity–distortion tradeoff was derived for this related model, but limited to the class of ergodic channels where the sequences of information densities are sure to converge.

While the generality of the presented “arbitrary/ergodic non-i.i.d”. models is appealing, the complexity of the expressions (both from an analytical perspective as well as in view of numerical evaluations) limits the utility of the results. An interesting approach is to consider larger (not only i.i.d.) classes of channels and source sequences for which the capacity–distortion tradeoff still has a relatively simple form.

In this spirit, the work in [86] characterized the capacity–distortion tradeoff of a class of channels that have previously been introduced and studied in the context of pure capacity calculations. This class of channels is also particularly interesting because the numerical calculation of capacity [87] as well as of the capacity–distortion tradeoff can be cast into the framework of Markov decision processes and thus solved using reinforcement learning (RL) as well as its many more advanced alternatives that have been introduced in recent years such as Q-learning, etc.

**Figure 5:**
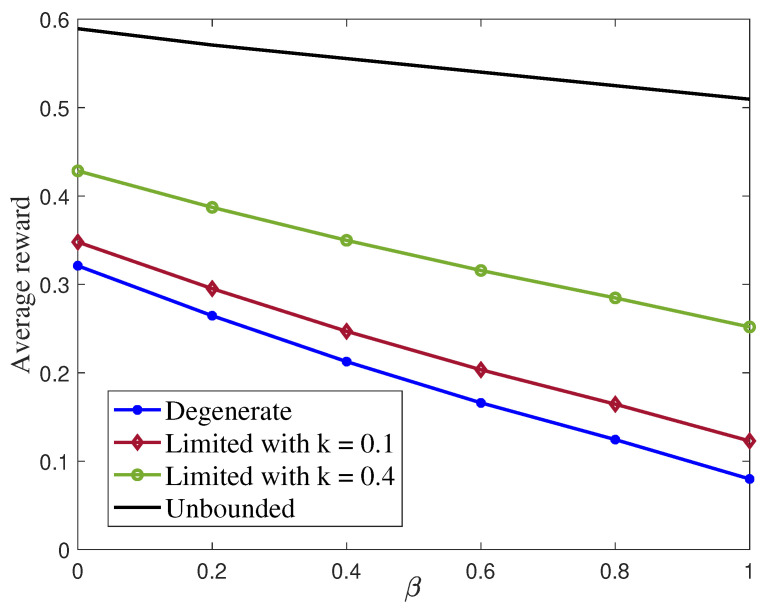
RL average reward composed of *β* times the information rate and (1−β) times the negative sensing distortion.

An RL approach was followed in [86] to evaluate the capacity–distortion tradeoff for a specific class of binary channels. Interestingly, the authors of [86] also analyzed the influence of the size of the state space considered in the RL approach, which corresponds to the memory in the coding strategy employed at the transmitter. Figure 5 plots a weighted sum between the information rate and the distortion in function of the weight factor β. It shows the performances achieved by four versions of the RL approach allowing for different sizes of the state spaces: a full state space; a highly-reduced state space that only allows to implement memoryless policies; and intermediate state spaces with sizes equal to 10% or 40% of the full state space and thus allowing the implementation of coding strategies with a limited amount of memory.

## 4. Sensing at the Rx (Rx-ISAC) with Sensing Distortion

In certain practical systems, sensing is performed at a device that differs from the radar-emitting device. Such situations are often referred to as bi-static radar. The information theory literature has considered various bi-static ISAC scenarios [21,61,62,88,89]. In this section, we focus on bi-static ISAC where the sensing task is performed at the communication Rx, and the sensing task is to estimate the target (state) up to a given distortion constraint.

The presented model captures one of the major challenges in bi-static ISAC, which is that the sensing terminal is a priori not aware of the channel input sequence and thus due to the memory in the channel input sequence, symbol-by-symbol estimators based solely on the observations are suboptimal. We shall see that when the sensing is performed at the communication Rx, this difficulty is easily solved by first decoding the data and reconstructing the input sequence, which contains all the memory in the system. In this case, a symbol-wise estimator based on this reconstructed input sequence and the observed sequence of channel outputs achieves minimum distortion (As we shall see in the section on Network ISAC, it is more complicated to characterize the optimal estimation strategy when the sensing terminal is not a priori required to decode all the transmitted data and codewords).

### 4.1. A Memoryless Model

**Figure 6:**
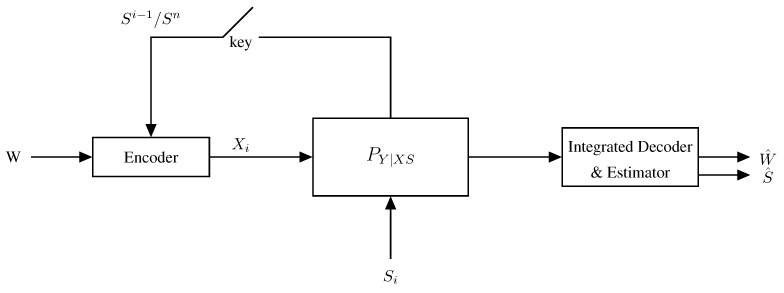
ISAC with Rx sensing.

We consider a similar memoryless model to that in the previous section. A single Tx wishes to communicate a message *W* to a single receiver over a state-dependent channel and the Rx aims to decode this message and at the same time also estimate the channel state sequence up to the allowed distortion, see Figure 6. In other words, the Rx applies a decoding function g(·) to its outputs to produce a message guess $\hat{W}=g\left(Y^{n}\right)$ and also an estimation function h(·) to produce the estimates ${\hat{S}}^{n}=h\left(Y^{n}\right)$.

To allow for a general model, we also include the models where the Tx knows the state sequence $S^{n}$ in one of the following ways:

- The Tx has no information about $S^{n}$;
- The Tx knows the entire sequence $S^{n}$ non-causally, i.e., before the entire transmission starts;
- The Tx knows $S^{n}$ in a strictly causal way, i.e., it learns $S_{i}$ only after channel use *i* and prior to channel use i+1;
- The Tx knows $S^{n}$ in a causal way, i.e., it learns $S_{i}$ just before channel use *i*.

Depending on the available state information, the Tx produces its time-*i* channel input either as a function of only the message *W* and the previous generalized feedback $Z^{i-1}$, or also in function of the entire state sequence $S^{n}$ (for the non-causal case), of the previous and the current state $S^{i}$ (for the causal case), or of the previous states $S^{i-1}$ only (for the strictly causal case).

The definition of the capacity–distortion function is analogous to Definition 1, but where encoding, decoding, and state estimation functions are as described above. Moreover, here, we do not consider cost constraints (which, however, could easily be included).

### 4.2. Capacity–Distortion Tradeoffs

We start with the model without state information at the Tx. In this case, the optimal estimator at the Rx is a symbol-by-symbol estimator based on the observed sequence of outputs and the decoded codeword, and the capacity–distortion tradeoff was characterized in [21].

**Theorem** **4.** *When the Tx has no knowledge about the state sequence* $S^{n}$**,* the capacity–distortion function is given by*

(26) $$\begin{array}{c} C_{\text{No-CSI}}:=\max_{P_{X}\in P_{D}}I\left(X;Y\right), \end{array}$$

*where*

(27) $$\begin{array}{ccc} P_{D} & = & \left\{P_{X}\;|\sum_{x\in X}P_{X}\left(x\right)E\left[d\left(S,{\hat{s}}^{*}\left(X,Y\right)\right)\right]\leq D\right\}, \end{array}$$

and ${\hat{s}}^{*}\left(\cdot ,\cdot \right)$ is the optimal estimator introduced in *(51)* with the feedback output Z replaced by the decoder output Y.

This capacity–distortion tradeoff for the setup where the Tx is not informed about the state sequence was also extended to a multi-access setup with multiple transmitters, see [88] and to a two-hop setup [90]. In the latter work, it is shown that a decode-(indirectly)-compress-and-forward strategy achieves the capacity–distortion function.

Consider next the scenarios where the Tx does learn the state sequence $S^{n}$ either causally or strictly causally, see [61,89]. In these cases, the Tx wishes to assist the Rx in the sensing task by conveying information about the state sequence to the receiver in the same spirit as it sends data. In other words, the Tx will compress the observed state sequence and send the compression information to the Rx, which then reconstructs the compressed version of the state. The Rx finally applies an optimal symbol-by-symbol estimator to this compressed sequence as well as to the decoded input codewords and the observed channel outputs.

In the case in which the Tx observes the state sequence only causally or even strictly causally, it has to employ a block Markov coding scheme, where in each block, it sends compression information about the state sequence from the previous block. Transmission of this compression information and of the data is performed using an optimal data communication scheme. Specifically, in the setup with strictly causal state information, a standard channel code is used that ignores the state information completely. For the setup with causal state information, the Tx has to resort to Shannon strategies, which have been shown to achieve capacity in these setups. Note that under Shannon strategies the channel inputs are generated symbolwise from an auxiliary codeword and the state sequence. The Rx thus does not have access to the channel inputs even when it decodes the codewords correctly. Nevertheless, it can be shown that the symbolwise estimator based on the decoded codewords and the observed channel output sequence achieves the optimal Rx sensing performance.

**Theorem** **5.**  (Theorem 2, [61]) *The capacity–distortion function for strictly causal state communication is*

(28) $$\begin{array}{c} C_{\text{Str-caus}.}\left(D\right)=\max_{P_{X}P_{U|XS}}\left(I(U,X;Y)-I(U,X;S)\right), \end{array}$$

where the maximum is over all laws $P_{X}P_{U|XS}$ such that $E[d\left(S,{\hat{s}}^{*}\left(U,X,Y\right)\right)]\leq D$, where (U,S,X,Y) are distributed according to $P_{S}P_{X}P_{U|XS}P_{Y|XS}$ and

(29) $${\hat{s}}^{*}\left(u,x,y\right)=\arg\min_{s^{\prime }\in \hat{S}}\sum_{s\in S}P_{S|UXY}\left(s|u,x,y\right)d\left(s,s^{\prime }\right).$$

*The capacity–distortion function for causal state communication is*

(30) $$\begin{array}{ccc} C_{\mathrm{Caus}.}\left(D\right) & = & \max\left(I(U,V;Y)-I(U,V;S)\right) \end{array}$$

where the maximum is over all laws $P_{V}P_{U|V,S}$ and functions x(v,s) such that $E[d\left(S,{\hat{s}}^{*}\left(U,V,Y\right)\right)]\leq D$, for (U,V,S,X,Y) distributed according to $P_{V}P_{S}P_{U|VS}1\left\{X=x\left(V,S\right)\right\}P_{Y|XS}$ and here,

(31) $${\hat{s}}^{*}\left(u,v,y\right)=\arg\min_{s^{\prime }\in \hat{S}}\sum_{s\in S}P_{S|UVY}\left(s|u,x,y\right)d\left(s,s^{\prime }\right).$$

In above expressions, the *U*- and *V*-auxiliaries stand for the auxiliary codewords. The subtracted mutual information terms arise because the Tx transmits compression information together with the data, and thus the rate of the compression information needs to be deduced from the total rate of communication that can be sustained from the Tx to the Rx.

When the Tx observes the state sequence non-causally, no block Markov strategies are necessary. Gel’fand–Pinsker (GP) coding [91], which achieves capacity for channels with non-causal state information at the Tx, is used to transmit the data and the compression information to the Rx. In GP coding, the channel inputs are again obtained as a function of auxiliary codewords and the state sequence. The Rx thus again cannot reconstruct the sequence of channel inputs, even after decoding the messages correctly. However, again, a symbol-by-symbol estimator based on the decoded codewords and the observed sequences achieves the optimal Rx sensing performance.

For this setup, with non-causal state information at the Tx, the exact capacity–distortion tradeoff is generally still an open problem, as only upper and lower bounds are known [89]. The work in [62] has characterized the exact capacity–distortion tradeoff in the case of a Gaussian model with mean squared error (MSE) distortion. This is, for a scenario where the time-*t* channel output is given by $Y_{t}=X_{t}+S_{t}+N_{t}$ for $X_{t}$ the channel input, $S_{t}$ a Gaussian state of variance Q; and $N_{t}$ a Gaussian noise of variance *N*, and $d\left(s,\hat{s}\right)={\left(s-\hat{s}\right)}^{2}$. For this case, the capacity–distortion tradeoff was derived in [62].

**Theorem** **6.** *The capacity–distortion tradeoff with non-causal state information at the Tx in the Gaussian case is given by:*

(32) $$\begin{array}{c} C_{\mathrm{Gaus}.}\left(D\right)=\max_{r\in [0,1]}\frac{1}{2}\log\left(1+\frac{rP}{N}\right), \end{array}$$

*where the maximum is over all values of r satisfying*

(33) $$\begin{array}{c} D\geq Q\frac{rP+N}{{\left(\sqrt{Q}+\sqrt{(1-r)P}\right)}^{2}+rP+N}. \end{array}$$

In the above theorem, the parameter r∈[0,1] indicates the fraction of the transmit power that the Tx uses for data transmission, i.e., to encode the message. The rest of the power, i.e., a fraction 1−r of the total power, is used to send channel state information (in an uncoded manner) to the Rx.

## 5. Network ISAC with Sensing Distortion

Modern communication systems are often multi-user and network-oriented, meaning that multiple Txs wish to simultaneously transmit data to multiple Rxs and some of these terminals have to accomplish sensing tasks. Characterizing the information-theoretic fundamental limits of multi-user network systems has been an active area of research for decades [92], and a vast majority of the systems still lack complete and computable characterizations of the fundamental performance limits, even when only data have to be transmitted, i.e., for systems without sensing tasks. Nevertheless, different interesting and insightful code constructions have been proposed for network communication systems and it has been shown that they perform reasonably close to the fundamental limits. In recent studies, researchers have introduced sensing tasks into these code constructions to obtain information-theoretic network ISAC schemes. Information-theoretic converse (infeasibility) results have also been derived for certain network ISAC scenarios. In this section, we shall review both network ISAC coding schemes and converse results. Note that network ISAC has also received significant attention in the signal processing and communication theory literature. We refer to [33,93] for these results.

We will start by reviewing a broadcast ISAC where communication is from a single Tx to multiple Rxs and the sensing is performed at the Tx. As we shall see, the sensing problem is the same as in the point-to-point communication scenario, and thus, the simple symbolwise estimator in (5) is optimal, so that the sensing problem and the communication problem “decouple" similarly to in the point-to-point case. The second scenario that we consider in this section is the multi-access ISAC problem with Tx sensing. Since the sensing task is accomplished at multiple distributed terminals with heterogeneous sensing information, this sensing problem is fundamentally different and allows for more complicated strategies, e.g., collaborative sensing strategies and the interactive exchange of sensing information between the different terminals. We shall present different ISAC coding schemes that perform the required communication tasks, and at the same time also exchange sensing information, thus allowing the implementation of collaborative sensing strategies. Similar strategies have also been proposed for device-to-device (D2D) communication (the two-way channel) and the interference channel (IC).

### 5.1. One-to-Many Communication (Broadcast Channels) with Tx Sensing

#### 5.1.1. The Memoryless Model

**Figure 7:**
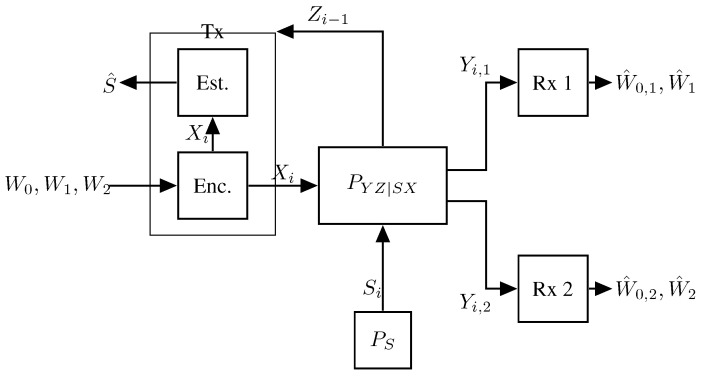
State-dependent broadcast channel with generalized feedback and state estimator at the Tx.

Consider the single-Tx two-Rx broadcast ISAC system, which is depicted in Figure 7.

Extensions to multiple Rxs follow standard techniques. The setup is similar to the single-user setup in Section 3.1, however, communication is to two distinct Rxs, 1 and 2. Specifically, the Tx wishes to communicate the rate-$R_{0}$ message $W_{0}$ to both Rxs, the rate-$R_{1}$ message $W_{1}$ to Rx 1, and the rate-$R_{2}$ message $W_{2}$ to Rx 2. The Tx thus produces inputs of the form $X_{i}=\phi_{i}\left(W_{0},W_{1},W_{2},Z_{1},\ldots ,Z_{i-1}\right)$, for i=1,…,n. The communication channel and the generalized feedback channel are governed by a state sequence $S^{n}$ that is i.i.d. according to $P_{S}$ and is jointly modeled by a stationary memoryless channel of transition probabilities $P_{Y_{1}Y_{2}Z|XS}\left(y_{1},y_{2},z|x,s\right)$ determining the outputs $\left\{Y_{1,i}\right\}$ at Rx 1, the outputs $\left\{Y_{2,i}\right\}$ at Rx 2, and the generalized feedback signals $\left\{Z_{i}\right\}$ at the Tx. Based on its observed channel outputs $Y_{k,1},\ldots ,Y_{k,n}$, each Rx k∈{1,2} produces the guesses ${\hat{W}}_{0,k}$ and ${\hat{W}}_{k}$ of the messages $W_{0}$ and $W_{k}$ using appropriate decoding functions $\left({\hat{W}}_{0,k},{\hat{W}}_{k}\right)=g_{k}\left(Y_{k,1},\ldots ,Y_{k,n}\right)$, and the Tx estimates the state sequences as $\left({\hat{S}}_{1},\ldots ,{\hat{S}}_{n}\right)=h\left(X_{1},\ldots ,X_{n},Z_{1},\ldots ,Z_{n}\right)$. As before, communication performance is measured in terms of decoding error probabilities and sensing performance in terms of expected average per-block distortion.

Accordingly, we have the following achievability definition, for a given bounded and non-negative distortion function d(·,·).

**Definition** **3.** *A rate–distortion tuple $\left(R_{0},R_{1},R_{2},D\right)$ is achievable if there exists a sequence (in n) of encoding, decoding, and state estimation functions such that*

(34a) $$\lim_{n\to \infty }Pr\left({\hat{W}}_{k}\neq W_{k}\;\mathrm{or}\;{\hat{W}}_{0,k}\neq W_{0}\right)=0,\;k\in \{1,2\},$$

(34b) $$\underset{n\to \infty }{\bar{\lim}}=\frac{1}{n}\sum_{i=1}^{n}E\left[d\left(S_{i},{\hat{S}}_{i}\right)\right]\leq D$$

*The closure of the set of all achievable rate–distortion tuples $\left(R_{0},R_{1},R_{2},D\right)$ is called the capacity–distortion region CD.*

**Remark** **2.** *The above model was considered in [63]. The authors of [64] considered a slightly different model where the state is composed of two components $S=(S_{1},S_{2})$, where each $S_{k}$ is revealed to the corresponding Rx and has to be estimated at the Tx up to a maximum allowed distortion $D_{k}$. As already mentioned for the point-to-point setup, receiver state information is included in the above model as a special case, by including the state information as part of the channel outputs. Similarly, since the state alphabet can be arbitrary but finite, in the above model, S can also be a pair of finite states $\left(S_{1},S_{2}\right)$. To be able to fully capture the setup and the results in [64] as special cases, it thus suffices to extend the above model from [63] to multiple distortion constraints, which can easily be done. The advantage of the model in [63] is that it is more general and allows the modeling of all kinds perfect or imperfect state information at the Rxs.*

#### 5.1.2. Results

The optimal estimator is again given by Lemma 1. That means, the optimal estimator is (irrespective of the choice of the encoding and decoding functions)

(35) $$\begin{array}{c} h_{k}^{*}\left(x^{n},z^{n}\right)=\left({\hat{s}}_{k}^{*}\left(x_{1},z_{1}\right),{\hat{s}}_{k}^{*}\left(x_{2},z_{2}\right),\ldots ,{\hat{s}}_{k}^{*}\left(x_{n},z_{n}\right)\right). \end{array}$$

where ${\hat{s}}^{*}\left(x,z\right)=\arg\min_{s^{\prime }\in \hat{S}}\sum_{s\in S}P_{S|XZ}\left(s|x,z\right)d\left(s,s^{\prime }\right)$ was defined in (5) and the conditional probability distribution $P_{S|XZ}\left(s|x,z\right)=\frac{P_{S}\left(s\right)P_{Z|SX}\left(z|s,x\right)}{\sum_{\tilde{s}\in S}P_{S}\left(\tilde{s}\right)P_{Z|SX}\left(z|\tilde{s},x\right)}$ again only depends on the channel and the state distribution.

Identification of this optimal estimator immediately allows a reduction of the problem of characterizing the capacity–distortion tradeoff region CD of the broadcast channel (BC) to the problem of identifying the set of communication rates that are achievable under a given constraint on the statistics of the channel input symbols. In this sense, we again notice a decoupling of the sensing problem and the communication problem for the BC as for the point-to-point channel. The communication problem needs to be solved under a constraint on the channel input statistics, but otherwise the sensing part does not interfere.

The pure communication problem over a memoryless BC with feedback is still open, and only inner and outer bounds are known for general channels. Notable exceptions are the classes of physically degraded BCs [94] (where feedback does not increase capacity) and other classes of BCs with states [7]. For these classes, with the help of the optimal estimator in (35), one can immediately characterize the capacity–distortion region CD, see [64]. For all other classes, the optimal estimator can be combined with the proposed coding schemes for BCs with feedback [2,3,4,5,6] the known infeasibility proofs (converses) to obtain inner and outer bounds on CD for general ISAC BCs.

The following inner and outer bounds on CD were reported in [63], see also [64].

**Theorem** **7** (Outer Bound)**.** *If $\left(R_{0},R_{1},R_{2},D\right)$ lies in CD, then there exist pmfs $P_{X},P_{U_{1}|X},P_{U_{2}|X}$ such that the random tuple $\left(U_{k},X,S,Y_{1},Y_{2},Z\right)∼P_{U_{k}|X}P_{X}P_{S}P_{Y_{1}Y_{2}Z|SX}$ satisfies the rate constraints*

(36a) $$R_{0}+R_{k}\leq I(U_{k};Y_{k}),\;k=1,2,$$

(36b) $$R_{0}+R_{1}+R_{2}\leq I(X;Y_{1},Y_{2}),$$

*and the average distortion constraints*

(37) $$E[d\left(S,{\hat{s}}^{*}\left(X,Z\right)\right)]\leq D.$$

**Proposition** **1** (Inner Bound)**.** *The capacity–distortion region CD includes all tuples $\left(R_{0},R_{1},R_{2},D\right)$ that for some choice of the auxiliaries $(U_{0},U_{1},U_{2},X,S,Y_{1},Y_{2},Z,V_{0},V_{1},V_{2})∼$$P_{U_{0}U_{1}U_{2}X}P_{S}P_{Y_{1}Y_{2}Z|SX}P_{V_{0}V_{1}V_{2}|U_{0}U_{1}U_{2}Z}$ satisfy inequalities *(38)* above and the distortion constraint *(37)*.*

(38a) $$R_{0}+R_{1}\leq I\left(U_{0},U_{1};Y_{1},V_{1}\right)-I\left(U_{0},U_{1},U_{2},Z;V_{0},V_{1}|Y_{1}\right)$$

(38b) $$R_{0}+R_{2}\leq I\left(U_{0},U_{2};Y_{2},V_{2}\right)-I\left(U_{0},U_{1},U_{2},Z;V_{0},V_{2}|Y_{2}\right)$$

(38c) $$\begin{array}{cc} R_{0}+R_{1}+R_{2}\leq & I\left(U_{1};Y_{1},V_{1}|U_{0}\right)+I\left(U_{2};Y_{2},V_{2}|U_{0}\right)+\min_{k\in \{1,2\}}I\left(U_{0};Y_{k},V_{k}\right)-I\left(U_{1};U_{2}|U_{0}\right)\\ & -I\left(U_{0},U_{1},U_{2},Z;V_{1}|V_{0},Y_{1}\right)-I\left(U_{0},U_{1},U_{2},Z;V_{2}|V_{0},Y_{2}\right)\\ & -\max_{k\in \{1,2\}}I\left(U_{0},U_{1},U_{2},Z;V_{0}|Y_{k}\right) \end{array}$$

(38d) $$\begin{array}{cc} 2R_{0}+R_{1}+R_{2}\leq & I\left(U_{0},U_{1};Y_{1},V_{1}\right)+I\left(U_{0},U_{2};Y_{2},V_{2}\right)-I\left(U_{1};U_{2}|U_{0}\right)\\ & -I\left(U_{0},U_{1},U_{2},Z;V_{0},V_{1}|Y_{1}\right)-I\left(U_{0},U_{1},U_{2},Z;V_{0},V_{2}|Y_{2}\right) \end{array}$$

**Figure 8:**
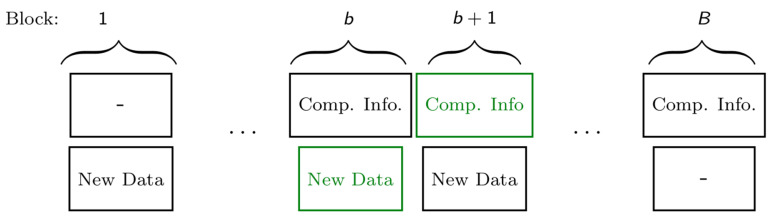
Block Markov coding structure.

The outer bound is obtained by considering a genie-aided system where Rx 2 observes not only the $Y_{2}$ outputs but also the $Y_{1}$ outputs, and by using the optimal estimator in (35). The inner bound is obtained by again combining this optimal estimator with the scheme in [5] for broadcast communication with generalized feedback. The scheme in [5] is based on a block Markov strategy (see Figure 8) where the Tx uses the generalized feedback signals in a block and its own transmitted signal in the same block to identify correlated compression information for both Rxs to improve their decoding. It then sends this update (compression) information in the following block, where the correlation allows the Tx to send part of the compression information as common information that is simultaneously useful for both Rxs, which is more efficient than sending individual information to the Rxs and thus is an improvement over no-feedback communication. Technically speaking, the common information is created using distributed compression techniques à la Gray-Wyner [29]. Decoding is performed backward, starting from the last block, where first the refinement information is decoded and then used to facilitate decoding of the previous block.

Both the inner bounds and the outer bounds are expressed with the help of *auxiliary random variables*. Examining the details of the proof of the inner bound in [64], the auxiliary random variables $U_{0},U_{1},U_{2}$ are easily identified with the different types of codewords used in the code construction. The auxiliary random variables $V_{0},V_{1},V_{2}$ are identified with codewords compressing the feedback signals and the auxiliary codewords corresponding to $U_{0},U_{1},U_{2}$. The $U_{k}$ auxiliary random variables however again point to a superposition structure given the Markov chains 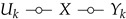.

More from a technical perspective, the auxiliary random variables allow the obtaining of inner and outer bounds that can be expressed as single-letter optimization problems. On the negative side, these optimization problems often still have high computational complexities.

#### 5.1.3. Example

Consider the physically degraded broadcast channel with binary input and output alphabets $X=Y_{1}=Y_{2}=\left\{0,1\right\}$ and two-bit state alphabet $S={\left\{0,1\right\}}^{2}$, i.e., the state *S* can be written as $S=(S_{1},S_{2})$ with binary $S_{1}$ and $S_{2}$. To describe the channel, let $Y_{k}^{\prime }=S_{k}\cdot X$ for each Rx k∈{1,2}, where the joint state pmf is:

(39) $$\begin{array}{cc} P_{S_{1}S_{2}}\left(s_{1},s_{2}\right) & =\left\{\begin{array}{cc} 1-q, & \mathrm{if}\;\left(s_{1},s_{2}\right)=\left(0,0\right)\\ 0, & \mathrm{if}\;\left(s_{1},s_{2}\right)=\left(0,1\right)\\ q\gamma , & \mathrm{if}\;\left(s_{1},s_{2}\right)=\left(1,1\right)\\ q(1-\gamma ) & \mathrm{if}\;\left(s_{1},s_{2}\right)=\left(1,0\right), \end{array}\right. \end{array}$$

for a real number γ,q∈[0,1]. The generalized feedback signals are $Z=(Y_{1}^{\prime },Y_{2}^{\prime })$ and the Rx outputs $Y_{k}=\left(Y_{k}^{\prime },S_{k}\right)$, which means that each Rx is informed of its corresponding state. Distortion is measured in terms of Hamming distortion between $S_{1}$ and an optimal estimator of $S_{1}$ based on (X,Z).

**Figure 9:**
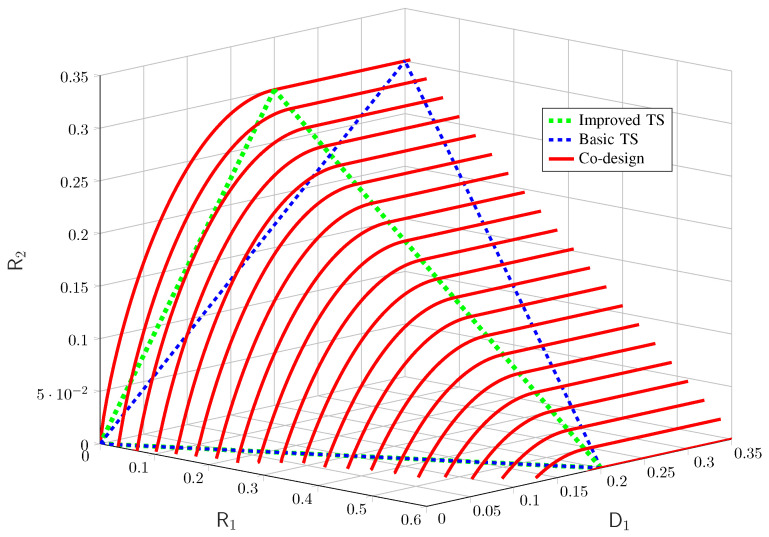
Capacity–distortion region for proposed example.

Note that $S_{2}$ is a degraded version of $S_{1}$, which together with the transition law, ensures the Markov chain 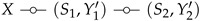 and the physically degradedness of the BC. For physically degraded BCs, the presented inner and outer bounds coincide [64] and thus, we can obtain the exact characterization of the capacity–distortion tradeoff of this example, which is shown numerically in Figure 9.

We observe a tradeoff between the two rates $R_{1}$ and $R_{2}$ and the permissible distortion *D*. Moreover, resource-sharing strategies are highly suboptimal as for the point-to-point case.

### 5.2. Multi-Access ISAC: Collaborative Sensing and Suboptimality of Symbolwise Estimators

This section reviews information-theoretic models for ISAC over multi-access channels (MAC). The first information-theoretic ISAC MAC scheme was proposed in [65] based on Willems’ coding scheme for data communication [95]. Willem’s scheme is again based on a block Markov strategy where in each block, the Txs not only send fresh data but also update information pertaining to the previous block. Again, the update information can be sent in a collaborative way, which renders the communication more efficient.

In [67,68], an improved collaborative ISAC scheme was proposed where the two Txs not only cooperate for the purpose of data transmission but also for the purpose of exchanging sensing information from one Tx to the others to allow the system to improve its sensing performance. More specifically, in [67,68], the two Txs exchange sensing information and data in each block, where the exchanged data is then retransmitted in the next block to improve the decoding performance at the Rx. Recently, in [66], the authors proposed a further improvement where the common update information sent by the two Txs not only consists of data but also includes sensing information, allowing the Rx to obtain better state information and thus improve the system’s decoding performance. (In the discussed models, the Rx has no sensing task, which, however, could easily be included.)

#### 5.2.1. The Memoryless Model

**Figure 10:**
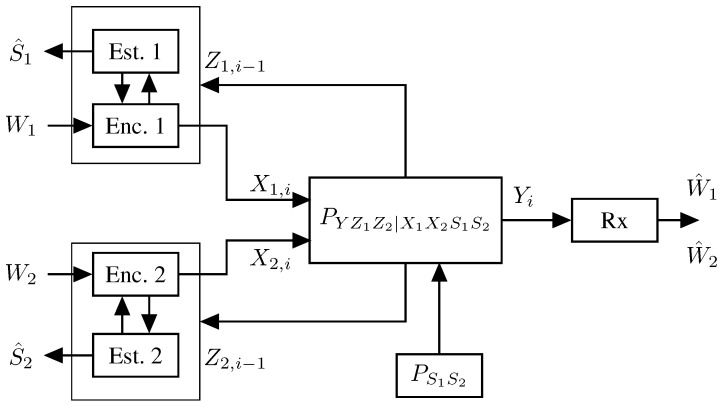
State-dependent discrete memoryless multi-access channel with sensing at the transmitters.

The model is similar to before, however, we now have two Txs and a single Rx, see Figure 10. Each Tx k∈{1,2} wishes to send a rate-$R_{k}$ message to the Rx and estimate a memoryless state sequence $\left\{S_{k,i}\right\}$. The sequence of pairs ${\left\{\left(S_{1,i},S_{2,i}\right)\right\}}_{i\geq 1}$ are i.i.d. according to a given joint pmf $P_{S_{1}S_{2}}$. The channel input–output relation is specified by the memoryless and stationary channel transition law $P_{YZ_{1}Z_{2}|S_{1}S_{2}X_{1}X_{2}}$. Based on the two messages $W_{1}$ and $W_{2}$ and the past generalized feedback signals $Z_{k,1},\ldots ,Z_{k,i-1}$, each Tx k∈{1,2} generates its time-i channel input as $X_{k,i}=\phi_{k,i}\left(W_{k},Z_{k,1},\ldots ,Z_{k,i-1}\right)$ and at the end of the communication, it estimates the state sequence as ${\hat{S}}_{k}^{n}=h_{k}\left(X_{k}^{n},Z_{k}^{n}\right)$. The estimated sequence ${\hat{S}}_{k}^{n}$ should match the state sequence $S_{k}^{n}$ up to distortion level $D_{k}$ when measured by a given per-symbol distortion function $d_{k}\left(\cdot ,\cdot \right)$. The receiver decodes both messages $W_{1}$ and $W_{2}$ based on its observed channel outputs as $\left({\hat{W}}_{1},{\hat{W}}_{2}\right)=g\left(Y^{n}\right)$.

**Definition** **4.** *A rate–distortion tuple $\left(R_{1},R_{2},D_{1},D_{2}\right)$ is called achievable in the setup above if there exists a sequence (in n) of encoding, decoding, and estimation functions such that*

(40a) $$\lim_{n\to \infty }Pr\left({\hat{W}}_{1}\neq W_{1}\;\mathrm{or}\;{\hat{W}}_{2}\neq W_{2}\right)=0$$

(40b) $$\underset{n\to \infty }{\bar{\lim}}\frac{1}{n}\sum_{i=1}^{n}E\left[d_{k}\left(S_{k,i},{\hat{S}}_{k,i}\right)\right]\leq D_{k},\;\mathrm{for}\;k\in \left\{1,2\right\}.$$

*In this multi-access ISAC setup, the closure of the set of all achievable tuples $\left(R_{1},R_{2},D_{1},D_{2}\right)$ is called the ISAC-MAC capacity–distortion region CD.*

Like the previous point-to-point and broadcast ISAC models, the above MAC model also includes scenarios with (perfect or imperfect) Rx channel state information as special cases. (The state information can simply be added as part of the output.) Note further that the above model also includes scenarios where the channel is governed by an internal i.i.d. state sequence $S^{n}$ of pmf $P_{S}$ and the states $S_{1}^{n},S_{2}^{n}$ are obtained from $S^{n}$ over an independent memoryless channel $P_{S_{1}S_{2}|S}$.

#### 5.2.2. Results

Determining the set of all achievable rates for the MAC with feedback is even open for only data communication, without the sensing task. Only a non-computable multi-letter expression is known in the general case [96]. Exceptions are the Gaussian MAC with perfect feedback [97] and a class of semi-deterministic MACs with one- or two-sided perfect feedback [98]. Various coding schemes [95,99,100,101] have been proposed, as well as an outer bound on the feedback capacity based on the dependence balance bound [102]. A recurrent theme in the presented coding schemes is that the feedback links to the two Txs allow to build up cooperation between the Txs. In fact, the feedback links establish a communication path from one Tx to the other, and the two Txs can thus (either implicitly as in [97] or explicitly as in [95,99,100,101]) align future channel inputs through cooperation, which amplifies the signals compared to the noise and allows for a better decoding performance at the Rx. As we shall see, the same idea is also key for proposing good MAC ISAC schemes.

There is a second fundamental idea that is required to achieve good ISAC MAC schemes, as we shall see in the following. It is inspired from and closely related to the works on multi-access communication over state-dependent channels where the Txs both have state information, see for example [103,104,105,106,107].

We first present infeasibility results for the ISAC multi-access problem. A first outer bound on the capacity distortion region CD was established in [65] and then improved in [66]. The outer bound in [66] is:

**Theorem** **8** (Outer Bound)**.** *The capacity–distortion region of the ISAC MAC CD is included in the set of all tuples $\left(R_{1},R_{2},D_{1},D_{2}\right)$ that for some pmf $P_{QQ_{Z}}P_{X_{1}X_{2}|QQ_{Z}}$ satisfy:*

(41) $$\begin{array}{cc} R_{1} & \leq I(X_{1};YZ_{1}Z_{2}|X_{2}QQ_{Z}), \end{array}$$

(42) $$\begin{array}{cc} R_{2} & \leq I(X_{2};YZ_{1}Z_{2}|X_{1}QQ_{Z}), \end{array}$$

(43) $$\begin{array}{cc} R_{1}+R_{2} & \leq I(X_{1}X_{2};YZ_{1}Z_{2}|QQ_{Z}), \end{array}$$

(44) $$\begin{array}{cc} R_{1}+R_{2} & \leq I(X_{1}X_{2};Y), \end{array}$$

*with the dependence balance constraint:*

(45) $$I\left(X_{1};X_{2}|QQ_{Z}\right)\leq I\left(X_{1};X_{2}|Z_{1}Z_{2}QQ_{Z}\right),$$

*and the sensing constraints:*

(46) $$\begin{array}{cc} D_{k} & \geq Ed(S_{k},{\hat{s}}^{*}\left(Z_{1},Z_{2},X_{1},X_{2}\right)),\;k\in \left\{1,2\right\}, \end{array}$$

(47) $$\begin{array}{cc} f_{k,R-D}\left(D_{k}\right) & \leq I\left(S_{k}X_{k^{\prime }};Z_{k}|X_{k}Q\right),\;k^{\prime },k\in \left\{1,2\right\},\;k^{\prime }\neq k, \end{array}$$

(48) $$\begin{array}{cc} f_{k,R-D}\left(D_{k}\right) & \leq I\left(S_{k};Z_{1}Z_{2}|X_{1}X_{2}Q\right),\;k\in \left\{1,2\right\}, \end{array}$$

*where $f_{k,R-D}\left(D_{k}\right)$ is the standard rate–distortion function of source $S_{k}$.*

*It suffices to consider Q and $Q_{Z}$ whose alphabets Q and $Q_{Z}$ have cardinalities satisfying $\left|Q|\cdot \right|Q_{Z}\left|\;\leq \;\right|X_{1}\left|\cdot \right|X_{2}|\;+\;3$.*

The outer bound is obtained by combining standard information-theoretic bounding steps with the following three key ideas: (1) providing Tx *k* also with Tx $k^{\prime }$ inputs and outputs $\left(X_{k^{\prime }}^{n},Z_{k^{\prime }}^{n}\right)$, for $k,k^{\prime }\in \left\{1,2\right\}$ and $k\neq k^{\prime }$, during the sensing task can only improve sensing performance and leads to constraint (46); (2) applying dependence balance considerations à la Hekstra and Willems [102] based on the pair of generalized feedback outputs $\left(Z_{1},Z_{2}\right)$ yields a valid constraint, see (45); and (3) the sensing distortion at Tx *k* cannot be smaller than the minimum sensing distortion in a joint source channel coding problem where the source $S_{k}^{n}$ is transmitted from Tx $k^{\prime }$ to *k*, see constraints (47) and (48). The former two key ideas were already exploited to derive the outer bound in [65]. Idea (3) was proposed in [66] and allows a strictly improved bound to be obtained.

**Figure 11:**
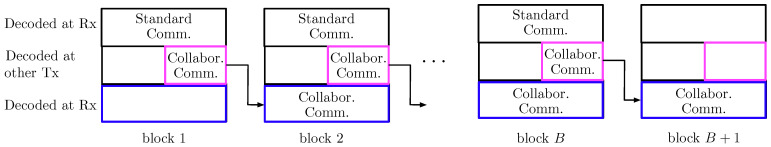
Block Markov strategy of Willems’ multi-access scheme with generalized feedback.

A first coding scheme (and thus achievability result) for the ISAC MAC was proposed in [65] based on Willems’ scheme for multi-access communication with feedback in [98]. The scheme is again based on a block Markov strategy where in each block, the two Txs send new independent data as well as common update information that will decoded at the Rx. More specifically, the scheme is illustrated in Figure 11 and each block consists of three layers, where the top-most layer is most difficult to decode and the lowest layer the easiest.

Both Txs send the same lowest layer, which thus can be transmitted in a cooperative manner, while the upper two layers are independent across the two transmitters. The details of the three layers are as follows:

- In the top layer, each Tx independently sends new data in each block. These data are decoded at the Rx only, following the backward decoding algorithm described later.
- In the middle layer, each Tx independently sends new data in each block. These data are decoded at the other Tx at the end of the block and at the Rx following the backward decoding algorithm described later.
- In the lowest layer, the two Txs cooperate and jointly resend the data sent by the two Txs in the middle layer of the previous block (recall that the medium layer data of the previous block has been decoded by the other Tx at the end of the previous block). These data are decoded at the Rx following the backward decoding algorithm described next.

The receiver decodes all transmitted data using a backward decoding procedure, starting from the last block. Specifically, for each block it decodes the data in the top and lowest layer, while it already is informed of the data sent in the middle layer, because it has decoded it in the previous step.

Each Tx *k* produces its state estimates ${\hat{S}}_{k}^{n}$ by using an optimal symbolwise estimator based on its own inputs $X_{k}^{n}$, its own observed generalized feedback signals $Z_{k}^{n}$, and also the middle-layer codeword symbols $U_{k^{\prime }}^{n}$ decoded from the other Tx $k^{\prime }\neq k$. It is clearly suboptimal for Tx *k* to estimate its state sequence $S_{k}^{n}$ simply based on its inputs $X_{k}^{n}$ and its feedback signals $Z_{k}^{n}$, and an improved performance can be obtained by attempting to decode also the codewords transmitted by the other Tx $k^{\prime }$.

**Figure 12:**
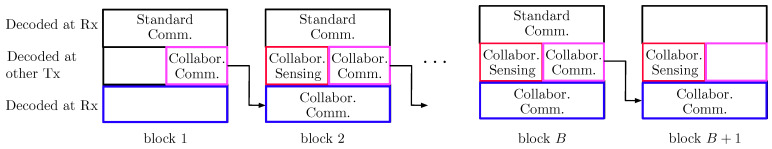
Block Markov strategy of the ISAC multi-access scheme in [68].

In [65], the sensing tasks and data communication tasks are thus still considered individually. A first joint approach was considered in [67,68], where sensing information was introduced to the coding scheme to allow for collaborative sensing; in other words, to allow each Tx to exploit sensing information available at the other Tx. On a technical level, this was enabled by having each Tx *k* compress the signals $U_{k^{\prime }}^{N}$, $X_{k}^{N}$, and $Z_{k}^{N}$ of a given block and send the compression information (described in bits) as additional information in the middle layer of the next codeword, see Figure 12. In this way, any of the two Txs can convey sensing information to the other Tx over the Tx-to-Tx path, because the information in the middle layer is decoded at the other Tx. The compression information is also decoded at the Rx, and used to improve decoding of the transmitted data. For the compression of the sensing information, the scheme in [67,68] uses implicit binning, i.e., the Tx as well as the Rx use their information about the compressed sequences from their own inputs and observations to reconstruct the sensing information. This allows the scheme to occupy less rate in the middle layer codewords and thus improve communication efficiency.

The other encoding and decoding steps are as in the scheme in [65] and described previously. Each Tx *k* now performs the sensing task by producing symbolwise estimates based on the triples $U_{k^{\prime }}^{N}$, $X_{k}^{N}$, and $Z_{k}^{N}$ and of the compression information obtained from the other Tx $k^{\prime }$.

The above coding schemes establish the following inner bound to the capacity–distortion tradeoff region CD [67,68].

**Theorem** **9.** *The capacity–distortion region CD of an ISAC MAC system includes any rate–distortion tuple $\left(R_{1},R_{2},D_{1},D_{2}\right)$ that for some choice of pmfs, $P_{U_{0}},P_{U_{1}|U_{0}},P_{U_{2}|U_{0}},P_{X_{1}|U_{0}U_{1}},P_{X_{2}|U_{0}U_{2}},$$P_{V_{1}|U_{0}U_{2}X_{1}Z_{1}},P_{V_{2}|U_{0}U_{1}X_{2}Z_{2}}$ satisfies Inequalities *(49)* below (where $\underline{U}:=\left(U_{0},U_{1},U_{2}\right)$)*

(49a) $$\begin{array}{cc} R_{k}\leq & I\left(U_{k};X_{k^{\prime }}Z_{k^{\prime }}|U_{0}U_{k^{\prime }}\right)+I\left(V_{k};X_{k^{\prime }}Z_{k^{\prime }}|\underline{U}\right)-I\left(V_{k};X_{k}Z_{k}|\underline{U}\right)\\ & +\min\{I\left(X_{k};Y|U_{0}X_{k^{\prime }}\right)+I\left(V_{k};X_{1}X_{2}Y|\underline{U}\right)+I\left(V_{k^{\prime }};X_{1}X_{2}YV_{k}|\underline{U}\right)-I\left(V_{k};X_{k}Z_{k}|\underline{U}\right),\\ & \;I\left(X_{1}X_{2};Y|U_{0}U_{k}\right)+I\left(V_{k};X_{1}X_{2}Y|\underline{U}\right)+I\left(V_{k^{\prime }};X_{1}X_{2}YV_{k}|\underline{U}\right)-I\left(V_{k^{\prime }};X_{k^{\prime }}Z_{k^{\prime }}|\underline{U}\right),\\ & \;I\left(X_{1}X_{2};Y|U_{0}\right)+I\left(V_{k};X_{1}X_{2}Y|\underline{U}\right)+I\left(V_{k^{\prime }};X_{1}X_{2}YV_{k}|\underline{U}\right)\\ & \;-I\left(V_{k};X_{k}Z_{k}|\underline{U}\right)-I\left(V_{k^{\prime }};X_{k^{\prime }}Z_{k^{\prime }}|\underline{U}\right),\;I\left(X_{k};YV_{1}V_{2}|\underline{U}X_{k^{\prime }}\right)\}\\ & k^{\prime },k\in \left\{1,2\right\},\;k\neq k^{\prime },\; \end{array}$$

(49b) $$\begin{array}{ccc} R_{1}+R_{2} & \leq & I\left(U_{2};X_{1}Z_{1}|U_{0}U_{1}\right)+I\left(V_{2};X_{1}Z_{1}|\underline{U}\right)-I\left(V_{2};X_{2}Z_{2}|\underline{U}\right)\\ & & +I\left(U_{1};X_{2}Z_{2}|U_{0}U_{2}\right)+I\left(V_{1};X_{2}Z_{2}|\underline{U}\right)-I\left(V_{1};X_{1}Z_{1}|\underline{U}\right)\\ & & +\min\{I\left(X_{1}X_{2};Y|U_{0}U_{2}\right)+I\left(V_{1};X_{1}X_{2}Y|\underline{U}\right)+I\left(V_{2};X_{1}X_{2}YV_{1}|\underline{U}\right)-I\left(V_{1};X_{1}Z_{1}|\underline{U}\right),\\ & & \;I\left(X_{1}X_{2};Y|U_{0}U_{1}\right)+I\left(V_{1};X_{1}X_{2}Y|\underline{U}\right)+I\left(V_{2};X_{1}X_{2}YV_{1}|\underline{U}\right)-I\left(V_{2};X_{2}Z_{2}|\underline{U}\right),\\ & & \;I\left(X_{1}X_{2};Y|U_{0}\right)+I\left(V_{1};X_{1}X_{2}Y|\underline{U}\right)+I\left(V_{2};X_{1}X_{2}YV_{1}|\underline{U}\right)\\ & & \;-I\left(V_{1};X_{1}Z_{1}|\underline{U}\right)-I\left(V_{2};X_{2}Z_{2}|\underline{U}\right),\\ & & \;I(X_{1}X_{2};YV_{1}V_{2}|\underline{U})\}\; \end{array}$$

(49c) $$\begin{array}{ccc} R_{1}+R_{2} & \leq & I\left(X_{1}X_{2};Y\right)+I\left(V_{1};X_{1}X_{2}Y|\underline{U}\right)-I\left(V_{1};X_{1}Z_{1}|\underline{U}\right)+I\left(V_{2};X_{1}X_{2}YV_{1}|\underline{U}\right)-I\left(V_{2};X_{2}Z_{2}|\underline{U}\right) \end{array}$$

*and for $k^{\prime },k\in \left\{1,2\right\}$ and $k^{\prime }\neq k$, the following satisfies*

(49d) $$I\left(U_{k};X_{k^{\prime }}Z_{k^{\prime }}|U_{0}U_{k^{\prime }}\right)+I\left(V_{k};X_{k^{\prime }}Z_{k^{\prime }}|\underline{U}\right)\geq I\left(V_{k};X_{k}Z_{k}|\underline{U}\right),$$

(49e) $$I\left(X_{1}X_{2};Y|U_{0}\right)+I\left(V_{1};X_{1}X_{2}Y|\underline{U}\right)+I\left(V_{2};X_{1}X_{2}YV_{1}|\underline{U}\right)\geq I\left(V_{1};X_{1}Z_{1}|\underline{U}\right)+I\left(V_{2};X_{2}Z_{2}|\underline{U}\right)$$

(49f) $$I\left(X_{k};Y|U_{0}X_{k^{\prime }}\right)+I\left(V_{1};X_{1}X_{2}Y|\underline{U}\right)+I\left(V_{2};X_{1}X_{2}YV_{1}|\underline{U}\right)\geq I\left(V_{k};X_{k}Z_{k}|\underline{U}\right).$$

*as well as the distortion constraints*

(50) $$\begin{array}{cc} D_{k} & \geq E[d_{k}\left(S_{k},\phi_{k}^{*}\left(X_{k},Z_{k},U_{k^{\prime }},V_{k^{\prime }}\right)\right], \end{array}$$

*for*

(51) $$\begin{array}{c} \phi_{k}^{*}\left(x_{k},z_{k},u_{k^{\prime }},v_{k^{\prime }}\right):=arg\min_{s^{\prime }\in \hat{S_{k}}}\sum_{s_{k}\in S_{k}}P_{S_{k}|X_{k}Z_{k}U_{k^{\prime }}V_{k^{\prime }}}\left(s_{k}|x_{k},z_{k},u_{k^{\prime }},v_{k^{\prime }}\right)\;d_{k}\left(s_{k},s^{\prime }\right). \end{array}$$

In the above theorem, the $U_{0}$ random variable stands for the common lowest-layer codeword of both Txs, $U_{1}$ and $U_{2}$ stand for the middle layer codewords of the two Txs, and $X_{1}$ and $X_{2}$ for the top codewords sent by the two Txs. The random variables $V_{1}$ and $V_{2}$ stand for the compression information produced at Tx 1 and 2, respectively. Accordingly, the previous achievable region in [65] is obtained as a special case from the above theorem by setting $V_{1}=V_{2}=$ constants.

**Figure 13:**
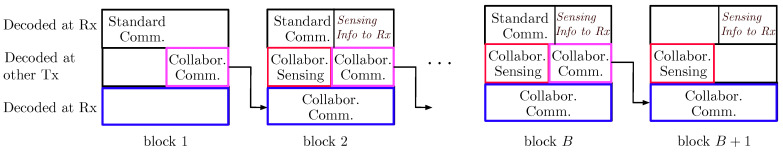
Block Markov strategy of the improved ISAC multi-access scheme in [66].

A further improvement was obtained in [66] by also adding sensing information to the lowest and the top codewords, see Figure 13. In other words, the two Txs jointly resend the two parts of exchanged compression information in a block in the next following block as part of the lowest codeword, and in each block, they individually add compression information to the top layer, which is not decoded at the other Tx but only at the Rx. Indeed, as already mentioned, the Rx can be interested in receiving compression information to improve its observations and thus decoding performance of the transmitted data.

It is rather straightforward to identify further ways of obtaining improved multi-access ISAC schemes. For example, one could add additional coding layers as in Marton coding in a way that the Rx is not required to decode all sensing information. In fact, in certain scenarios, sensing information is useless at the Rx, and moreover, the Rx has worse decoding capabilities than the Txs. Moreover, joint source-channel coding methods could be applied for the transmission of sensing information. In fact, the sensing information sent at the two Txs are correlated and it is well known that in such a scenario, a joint source-channel coding approach can achieve improved performances. In an upcoming section, we briefly discuss a joint source-channel coding approach for the two-way channel, i.e., for device-to-device (D2D) communication.

#### 5.2.3. Example

The following example shows the improvement in Theorem 9 over the previous scheme in [65]. As mentioned, a further improvement is achieved by the scheme in [66].

**Example** **2.** *Consider binary noise, states, and channel inputs $B_{0},B_{k},S_{k},X_{k}\in \left\{0,1\right\}$. The noise to the Rx $B_{0}$ is Bernoulli-$t_{0}$, and $B_{k}$, the noise on the feedback link to Tx k is Bernoulli-$t_{k}$. All noise sources are independent and also independent of the states $S_{1},S_{2}$, which are i.i.d. Bernoulli-$p_{s}$. We can then describe the channel as*

(52) $$\begin{array}{cc} Y^{\prime }=S_{1}X_{1}+S_{2}X_{2}+B_{0}, & \;Y=\left(Y^{\prime },S_{1},S_{2}\right), \end{array}$$

(53) $$\begin{array}{cc} \;Z_{1}=S_{1}X_{1}+S_{2}X_{2}+B_{1}, & \;Z_{2}=S_{1}X_{1}+S_{2}X_{2}+B_{2} \end{array}$$

*In this example, the Rx has perfect channel state information and the Hamming distance is considered as a distortion measure: $d\left(s,\hat{s}\right)=s⊕\hat{s}$.*

**Figure 14:**
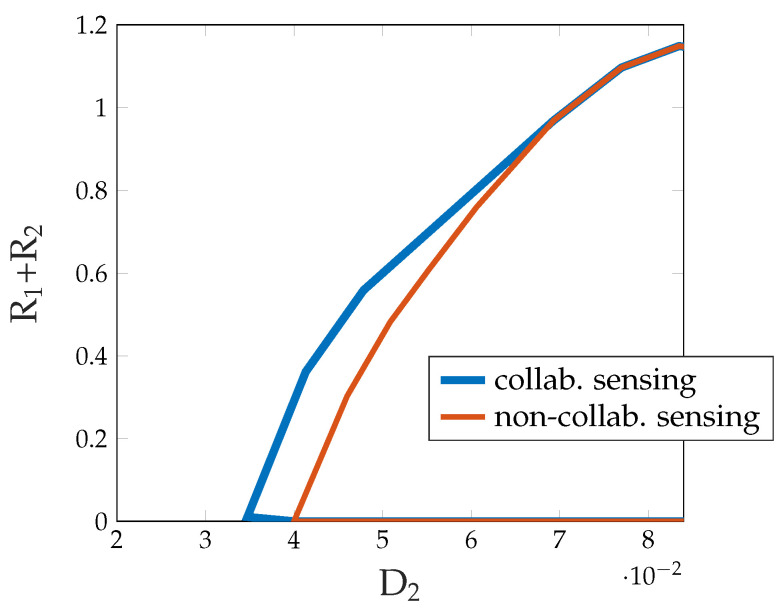
Sum-rate distortion tradeoff in Example 2 achieved without and with collaborative sensing, for given channel parameters $p_{s}=0.9$, $t_{0}=0.3$, $t_{1}=0.1$ and $t_{2}=0.1$.

*Figure 14 shows the maximum sum-rate $R_{1}+R_{2}$ as a function of distortion $D_{2}$ achieved by Theorem 9 (with collaborative sensing) and the region in [65] without collaborative sensing where $V_{1}=V_{2}=$ constants. Both curves are strictly concave and thus are improvements over classic time- and resource-sharing strategies. The minimum distortions achieved are $D_{2,\min}=0.035$ with collaborative sensing and $D_{2,\min}=0.04$ without.*

Similar ISAC coding ideas were also proposed for the interference channel (IC) where two Txs communicate to two Rxs [108]. The idea is to use block Markov coding as for the MAC and that the two Txs compress and convey sensing information in addition to the cooperative data communication in previous blocks. The corresponding set of achievable rate-distortion tuples can be found in [108].

### 5.3. Device-to-Device (D2D) Communication (Two-Way Channel)

**Figure 15:**
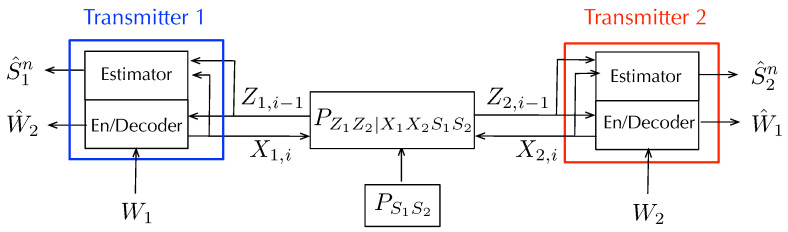
State-dependent discrete memoryless two-way channel with sensing at the terminals.

In addition to ISAC multi-access systems, the authors of [68] also studied the related two-way channel, i.e., device-to-device (D2D) communication. The D2D setup is illustrated in Figure 15 and is similarly defined to the MAC, except that message $W_{1}$ has to be decoded at Tx 2 and message $W_{2}$ at Tx 1. There is thus no receiver terminal. The capacity–distortion region CD is defined in analogy to the MAC setup.

The capacity region for D2D data communication (without a sensing task), and thus the optimal coding scheme, is still open in general. Various inner and outer bounds on the capacity region have been proposed. Han [109] and Kramer [110] proposed schemes that correlate the inputs of the two terminals in a block-fashion, while for Han’s coding scheme the correlation ensures a stationary distribution of the inputs and outputs across the blocks and thus still allows for single-letter rate-expressions, Kramer has to resort to multi-letter rate expressions based on directed mutual information. An interesting outer bound on the capacity region was proposed by Hekstra and Willems [102] again based on the dependence-balance idea, similar to the MAC with feedback.

The authors of [68] proposed two coding schemes for the ISAC D2D problem. The idea of the first scheme is to extend Han’s D2D coding scheme similar to the way the authors of [68] extended Willems’ scheme for the MAC. That means, the two terminals generate compression information, which they convey to the other Tx as part of the indices sent in the data communication scheme. A second, more advanced coding scheme based on joint source-channel coding, was also proposed in [68]. In this second scheme, the compression information is not just transmitted by means of indices sent instead of data but also by correlating the channel inputs with the sensing information (i.e., the compression codewords), as is typically done in hybrid coding [28]. This allows the two terminals to directly transfer the correlation of the sensing information to the channel inputs, which often allows for improved decoding performances at the two Txs.

**Theorem** **10** (Inner Bound via Joint Source-Channel Coding)**.** *The capacity–distortion region of the D2D ISAC problem CD contains all rate–distortion quadruples $\left(R_{1},R_{2},D_{1},D_{2}\right)$ for which there exists a choice of the pmf $P_{U_{1}^{\prime }U_{2}^{\prime }Z_{1}Z_{2}X_{1}X_{2}U_{1}U_{2}}$ and functions $f_{1}$ and $f_{2}$ satisfying the stationarity condition *(54)* on top of this page*

(54) $$\begin{array}{l} P_{U_{1}^{\prime }U_{2}^{\prime }Z_{1}Z_{2}X_{1}X_{2}U_{1}U_{2}}\left(u_{1}^{\prime },u_{2}^{\prime },z_{1},z_{2},x_{1},x_{2}\right)\\ \;=\sum_{{\tilde{u}}_{1},{\tilde{u}}_{2},{\tilde{x}}_{1},{\tilde{x}}_{2},{\tilde{z}}_{1},{\tilde{z}}_{2}}P_{U_{1}^{\prime }|X_{1}Z_{1}{\tilde{U}}_{1}{\tilde{X}}_{1}{\tilde{Z}}_{1}}\left(u_{1}^{\prime }|u_{1},x_{1},z_{1},{\tilde{u}}_{1},{\tilde{x}}_{1},{\tilde{z}}_{1}\right)P_{U_{2}^{\prime }|X_{2}Z_{2}{\tilde{U}}_{2}{\tilde{X}}_{2}{\tilde{Z}}_{2}}\left(u_{2}^{\prime }|x_{2},z_{2},u_{2},{\tilde{u}}_{2},{\tilde{x}}_{2},{\tilde{z}}_{2}\right)\\ \;\cdot P_{Z_{1}Z_{2}|X_{1}X_{2}}\left(z_{1},z_{2}|x_{1},x_{2}\right)1\left\{x_{1}=f_{1}\left(u_{1},{\tilde{u}}_{1},{\tilde{x}}_{1},{\tilde{z}}_{1}\right)\right\}1\left\{x_{2}=f_{2}\left(u_{2},{\tilde{u}}_{2},{\tilde{x}}_{2},{\tilde{z}}_{2}\right)\right\}\\ \;\cdot P_{U_{1}^{\prime }U_{2}^{\prime }Z_{1}Z_{2}X_{1}X_{2}U_{1}U_{2}}\left(u_{1},u_{2},{\tilde{z}}_{1},{\tilde{z}}_{2},{\tilde{x}}_{1},{\tilde{x}}_{2},{\tilde{u}}_{1},{\tilde{u}}_{2}\right), \end{array}$$

*and so that the following two rate constraints*

(55) $$\begin{array}{ccc} R_{k} & \leq & I\left({\tilde{U}}_{k};X_{k^{\prime }},Z_{k^{\prime }},{\tilde{U}}_{k^{\prime }},{\tilde{X}}_{k^{\prime }},{\tilde{Z}}_{k^{\prime }}\right)-I\left(U_{k};X_{k},Z_{k},{\tilde{U}}_{k},{\tilde{X}}_{k},{\tilde{Z}}_{k}|X_{k^{\prime }},Z_{k},{\tilde{U}}_{k^{\prime }},{\tilde{X}}_{k^{\prime }},{\tilde{Z}}_{k^{\prime }}\right),\\ & & \;k,k^{\prime }\in \left\{1,2\right\},\;k^{\prime }\neq k, \end{array}$$

*and the two distortion constraints in*

(56) $$\begin{array}{ccc} E[d_{k}\left(S_{k},\;\phi_{2,k}^{*}\left(U_{k^{\prime }}^{\prime },X_{k^{\prime }},Z_{k^{\prime }},U_{k^{\prime }},X_{k},Z_{k},{\tilde{U}}_{k},{\tilde{X}}_{k},{\tilde{Z}}_{k},{\tilde{U}}_{k^{\prime }}\right)\right)] & \leq & D_{k},\;k,k^{\prime }\in \left\{1,2\right\},\;k^{\prime }\neq k, \end{array}$$

*hold.*

## 6. Secrecy of ISAC Systems

Secrecy is a major concern in ISAC systems, both for the communication and the sensing tasks. Depending on the applications, adversaries should not be able to learn the transmitted data and/or infer information about the sensing targets. The information-theoretic literature has mostly studied the problem of ensuring secrecy of messages [69,85,111,112], but first results also exist to ensure secrecy of sensing information [70]. The model is important because ISAC systems enable the surveillance of the environment, and in many scenarios, it is crucial to prevent unauthorized access to user or channel information. In this section, we review both these lines of work.

### 6.1. Secrecy of the Message: The Memoryless Model

**Figure 16:**
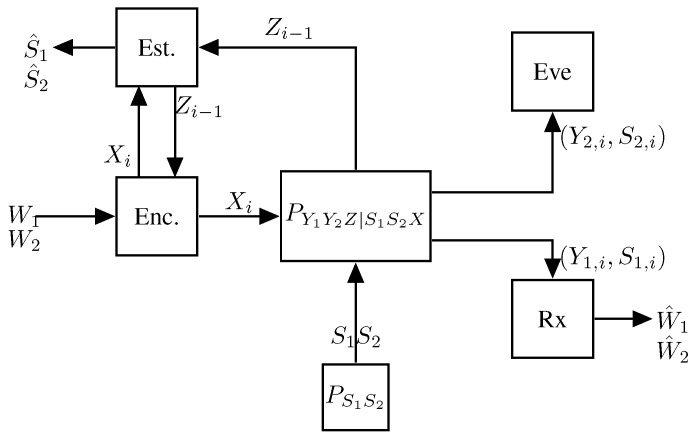
ISAC model under partial secrecy, where only $W_{2}$ should be kept secret from Eve.

Based on the memoryless ISAC model in [56], a wiretap equivalent was introduced in [69], see Figure 16. In this model, communication needs to be such that the eavesdropper cannot learn part of the message, which is formalized by the requirement that the equivocation between this specific message part and Eve’s observations should vanish for large blocklengths (This requirement is also known as strong secrecy in the information-theoretic literature).

Formally, the problem is defined with a Tx, a legitimate Rx, and an eavesdropper (Eve). The Tx aims to communicate a pair of messages $\left(W_{1},W_{2}\right)$ of rates $R_{1}$ and $R_{2}$ to the legitimate receiver, in a way that Eve cannot learn any information about the message $W_{1}$. (There is no constraint on how much Eve learns about the other message $W_{2}$. Communication is over a memoryless stationary state-dependent channel $P_{Y_{1}Y_{2}Z|SX}$ where *X* is the channel input, $S_{1}$ and $S_{2}$ are the states, $Y_{1}$ the outputs at the legitimate receiver, $Y_{2}$ the outputs at the eavesdropper, and *Z* The generalized feedback. The state sequences $\left\{\left(S_{1,i},S_{2,i}\right)\right\}$ are assumed i.i.d. according to the given law $P_{S_{1}S_{2}}$, and the transmitter creates the time-*i* channel inputs as $X_{i}=\phi_{i}\left(W_{1},W_{2},Z_{i-1}\right)$ using some appropriate encoding function $\phi_{i}$. At the end of the communication, the Tx estimates the state sequence as ${\left({\hat{S}}_{1},{\hat{S}}_{2}^{n}\right)}^{n}=h\left(X^{n},Z^{n}\right)$. The receiver decodes the two messages as $\left({\hat{W}}_{1},{\hat{W}}_{2}\right)=g\left(Y_{1}^{n},S_{1}^{n}\right)$ using an appropriate encoding function. The goal of the communication is that decoding error probability vanish asymptotically, that the reconstructed state sequence matches the correct state up to a given distortion constraints $D_{1}$ and $D_{2}$ under given per-symbol distortion measures $d_{1}\left(\cdot ,\cdot \right)$ and $d_{2}\left(\cdot ,\cdot \right)$, and that Eve learns nothing about message $W_{2}$ from her observations $Y_{2}^{n}$ and $S_{2}^{n}$.

**Definition** **5.** *A secrecy–rate–distortion tuple $\left(R_{1},R_{2},D_{1},D_{2}\right)$ is achievable if it is possible to find a sequence (in the blocklength n) of encoding, decoding, and estimation functions satisfying*

(57) $$\begin{array}{ccc} \lim_{n\to \infty }\mathrm{Pr}\left[W_{k}\neq {\hat{W}}_{l}\right] & = & 0,\;k\in \{1,2\}, \end{array}$$

(58) $$\begin{array}{ccc} \lim_{n\to \infty }I\left(W_{2};Y_{2}^{n},S_{2}^{n}\right) & = & 0, \end{array}$$

(59) $$\begin{array}{ccc} \underset{n\to \infty }{\bar{\lim}}E\left[d\left(S_{k}^{n},{\hat{S}}_{k}^{n}\right)\right] & \leq & D_{k},\;k\in \left\{1,2\right\}. \end{array}$$

*The closure of the set of all achievable secrecy–rate–distortion tuples $\left(R_{1},R_{2},D_{1},D_{2}\right)$ is called the secrecy–capacity–distortion region SCD.*

Here, we chose to present the slightly restricted model where the Rxs learn the two state sequences, thus not allowing for no or only imperfect state information. A more general model can, however, easily be obtained similar to the models presented in the previous sections. The reason for considering this special case is that in the following, we will limit to the special case where the Tx observes perfect output feedback, i.e., $Z=(Y_{1},Y_{2})$ without the two states, which does not allow the incorporation of arbitrary channel state information distributions at the Tx and the Rx/Eve.

### 6.2. Secrecy of Messages: Results

Most of the results have been derived under the assumption of perfect feedback from both the Rx and Eve, i.e., $Z=(Y_{1},Y_{2})$ [69]. Only the outer bounds in [69] apply for a slightly more general scenario where Z is a noisy version of $\left(Y_{1},Y_{2}\right)$.

Note that the optimal estimator at the Tx is the same as in the setup without a secrecy constraint, see (5). In the case of perfect output feedback $Z=(Y_{1},Y_{2})$ and two states, these optimal estimators are:

(60) $$\begin{array}{ccc} {\hat{s}}_{k}^{*}\left(x,y_{1},y_{2}\right) & = & \arg\min_{s^{\prime }\in {\hat{S}}_{k}}\sum_{s\in S_{k}}P_{S_{k}|XY_{1}Y_{2}}\left(s|x,y_{1},y_{2}\right)d_{k}\left(s,s^{\prime }\right),\;k\in \left\{1,2\right\}. \end{array}$$

Combined with these optimal estimators, the output statistics of random binning (OSRB) proof technique [113] allows the following result to be achieved [69].

**Theorem** **11** (Inner Bound)**.** *The secrecy–capacity–distortion region SCD contains all secrecy–rate–distortion tuples $\left(R_{1},R_{2},D_{1},D_{2}\right)$ that satisfy the following inequalities for some pmf $P_{UVX}$:*

(61) $$R_{1}\leq I(U;Y_{1}S_{1})$$

(62) $$R_{2}\leq \min\{{\left[I\left(V;Y_{1}|S_{1}U\right)-I\left(V;Y_{2}|S_{2}U\right)\right]}^{+}+H\left(Y_{1}S_{1}|Y_{2}S_{2}V\right),\;\left(I\left(V;Y_{1}|S_{1}\right)-R_{1}\right)\}$$

(63) $$D_{k}\geq E\left[d_{k}\left(S_{k},{\hat{s}}_{k}^{*}\left(X,Y_{1},Y_{2}\right)\right)\right],\;k\in \left\{1,2\right\}.$$

**Theorem** **12** (Outer Bound)**.** *The secrecy–capacity–distortion region SCD is included in the union over all joint distributions $P_{UVX}=P_{UV}P_{X|V}$ of all rate tuples $\left(R_{1},R_{2},D_{1},D_{2}\right)$ satisfying. The bounds are slightly simpler and stronger than the bounds in [69] and can be proved using similar steps.*

(64) $$R_{1}+R_{2}\leq I(V;Y_{1}|S_{1}),$$

(65) $$R_{2}\leq I(V;Y_{1}S_{1}|Y_{2}S_{2}),$$

(66) $$D_{k}\geq E\left[d_{k}\left(S_{k},{\hat{s}}_{k}^{*}\left(X,Y_{1}Y_{2}\right)\right)\right],\;k\in \left\{1,2\right\},$$

*One can limit V to $|V|\leq \min\{|X|,\;|Y_{1}||S_{1}|,\;|Y_{2}||S_{2}|\}+1$.*

The above results assume that only a part of the message (namely $W_{2}$) has to be kept secure from Eve. Corresponding results where all messages have to be kept secure are easily obtained by setting $R_{1}=0$ and interpreting $R_{2}$ as the total rate of all communicated messages.

Above inner and outer bounds do not coincide in the general case. They do in the case of degraded channels where $P_{Y_{2}S_{2}|XS_{1}Y_{1}}=P_{Y_{2}S_{2}|Y_{1}S_{1}}$ and reversely degraded channels where $P_{Y_{1}S_{1}|XS_{2}Y_{2}}=P_{Y_{1}S_{1}|Y_{2}S_{2}}$. They have also been specialized to several interesting and practical channels. In particular, the results for the Gaussian fading examples are worth being mentioned for binary states [111] as well as for Rayleigh fading states [112].

Finally, note that a finite blocklength analysis for ISAC with security constraints has been performed in [85].

### 6.3. Secrecy of Data and Sensing Information

In [70], not only the message (data) has to be kept secure from an external eavesdropper but also the channel state sequence $S^{n}$. Depending on the channel, Eve will always learn about the channel state; however, it is required that this knowledge stays beyond a given threshold. In other words, the Tx has to choose transmission strategies in a way that not too much information is leaked about the sensing target. In a practical application this could mean that the Tx has to restrict itself to beamforming strategies where it points its beam towards a given direction.

**Figure 17:**
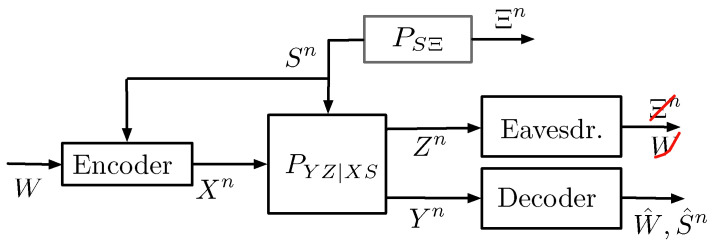
ISAC model with state information at the Tx and secrecy constraints on messages and states.

To make the problem more interesting from a technical point of view, the authors in [70] focused on the setup in Figure 17 where the Tx learns the state sequence $S^{n}$ in a non-causal manner (i.e., before transmission starts) and the sensing is performed at the Rx. An external eavesdropper is not allowed to learn any information about the message nor the sequence $\Xi^{n}$, which is obtained by passing the state sequence $S^{n}$ through a memoryless channel $P_{\Xi |S}$ independent of the message and the communication channel. Note that the channel $P_{\Xi |S}$ needs to be carefully chosen in the model to reflect the desired security constraint. For example, it could select a part of the state if *S* is bipartite $S=(S_{1},S_{2})$ and only one of these states needs to be kept secret, or it could implement a function Ξ=ν(S) when only certain characteristics of the target have to be kept secret. In general, the setup in [70] allows the modeling of any stochastic relationship between the state *S* and the part that needs to be kept secret Ξ.

In the setup of this section, there is only one message *W* of rate *R* and the Tx produces its channel inputs as $X_{i}=\phi_{i}\left(W,S^{n}\right)$, for $S^{n}$ the i.i.d. state sequence following a given pmf $P_{S}$. The channel outputs $Y^{n}$ observed at the legitimate Rx and $Z^{n}$ observed at the eavesdropper are produced from inputs and states according to a given stationary and memoryless channel law $P_{YZ|XS}$. Based on the observed outputs $Y^{n}$, the Rx decodes the message as $\hat{W}=g\left(Y^{n}\right)$ and produces an estimate of the state sequence ${\hat{S}}^{n}=h\left(Y^{n}\right)$. The goal of the Tx is to find an encoding strategy for which the Rx can decode with arbitrary small probabilities of error and reconstruct the state sequence with desired distortion *D* but such that the eavesdropper does not learn about the related sequence $\Xi^{n}$ nor the message *W*. This leads to the following definition of achievability.

**Definition** **6.** *A rate–distortion pair (R,D) is called securely achievable if there exists a sequence (in n) of encoding, decoding, and estimation functions such that*

(67a) $$\lim_{n\to \infty }\mathrm{Pr}\left[\hat{W}\neq W\right]=0$$

(67b) $$\lim_{n\to \infty }I\left(W,\Xi^{n};Z^{n}\right).=0$$

(67c) $$\underset{n\to \infty }{\bar{\lim}}\frac{1}{n}\sum_{i=1}^{n}E\left[d\left(S_{i},{\hat{S}}_{i}\right)\right]\leq D.$$

The following inner bound was proved in [70].

**Theorem** **13.** *For any pmf $P_{UVX|S}$ so that for the associated tuple $\left(S,\Xi ,U,V,X,YZ\right)∼P_{S}P_{\Xi |S}P_{UVX|S}P_{YZ|XS}$, the random variable *Ξ* is independent of the pair (U,Z) and any function g(·) on appropriate domains, all pairs $\left(R_{M},D\right)$ satisfying the following inequalities*

(68) R≤I(UV;Y)−I(UV;S)

(69) R≤I(V;Y∣U)−I(V;ΞZ∣U)+min{0,I(U;Y)−I(U;S)}

*and*

(70) $$\begin{array}{ccc} D & \leq & E[d(S,g(U,V,Y))] \end{array}$$

*are securely achievable.*

The above achievability result is based on the following coding scheme. A two-level superposition code with cloud-center codewords $U^{n}$ and satellite codewords $V^{n}$ is considered. The Tx uses the $U^{n}$-codewords to describe information about the state sequence $S^{n}$ to the receiver, where this cloud-center codeword can also be decoded by the eavesdropper. It further uses the $V^{n}$-codewords to send more refined information about $S^{n}$ as well as the message *W* to the Rx. The Rx decodes both the $U^{n}$ and $V^{n}$ codewords to recover the transmitted message *W*. It also reconstructs the state sequence based on the two decoded codewords and its own observed sequence of channel outputs. Security of the scheme against the external eavesdropper is obtained by choosing the $U^{n}$-codewords so that the decoded does not reveal information about the $\Xi^{n}$-sequence (because the $U^{n}$-codeword is also decoded by the eavesdropper). In fact, in the construction, only the $V^{n}$-codeword can contain information about $\Xi^{n}$ and *W*, and they are chosen of sufficiently high rate so that the eavesdropper cannot decode them.

The theorem includes several interesting special cases. When *Z* is independent of the input-state pair (X,S), the setup reduces to the setup without secrecy constraint studied in [89], in which case Theorem 13 can be simplified by choosing U= const. On a different note, when the entire state *S* has to be kept secret, Ξ=S, then *U* has to be chosen independently of *S* and thus I(U;S)=0 and the minimum in the right-hand side of (69) evaluates to 0. Moreover, for Ξ=S the right-hand side of (68) is larger than the right-hand side of (69) because I(V;SZ|U)≥I(V;S|U). Thus, for Ξ=S, Constraint (68) is less stringent than Constraint (69) where *U* only plays the role of a convexification random variable.

Comparing the results where both message and state have to be kept secret with the results with no secrecy constraint is applied, the price for the double state and message secrecy in the proposed scheme seems to be independence of *S* with *Z* and the rate reduction of I(V;Z|S)=I(SV;Z), see ([70], Corollaries 1–3).

**Example** **3.** *From [70]. Consider the interesting examples with Gaussian channels*

(71) $$Y_{i}=X_{i}+S_{i}+N_{i},$$

(72) $$Z_{i}=aX_{i}+bS_{i}+N_{e,i},$$

*for some given parameters a,b and $\left\{N_{i}\right\}$ and $\left\{N_{e,i}\right\}$ memoryless standard Gaussian noise sequences. Let further*

**Figure 18:**
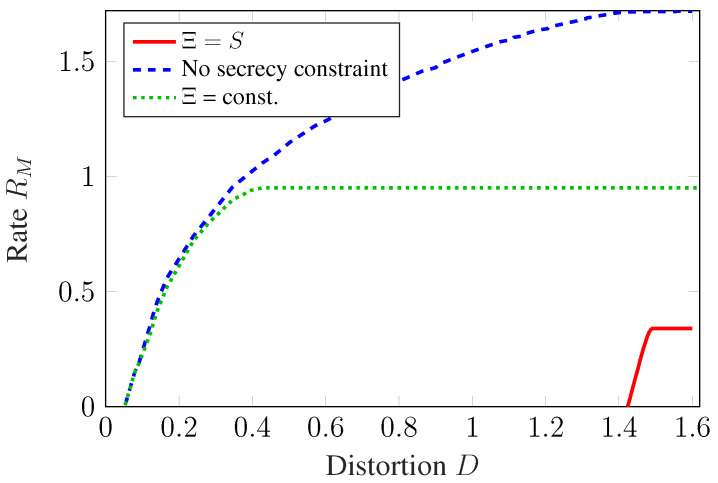
Comparison of the achievable rate–distortion tradeoffs under different secrecy constraints.

(73) Ξ=S+A,

*for $A∼N(0,\sigma_{A}^{2}\geq 0)$ independent of all other r.vs. This setup covers the scenario where the entire state sequence has to be kept secret, with the choice $\sigma_{A}^{2}=0$, and (with a slight abuse of notation) the scenario where the state does not have to be kept secret at all, with the choice $\sigma_{A}^{2}\to \infty$. The following Figure 18 shows an achievable set of rate–distortion pairs according to above Theorem 13 for a=0.7 and b=0.3 and S∼N(0,3) and with a input block power constraint of P=30. The set of achievable rate–distortion pairs is provided without any secrecy constraints neither on messages nor state, with full secrecy constraints on both (Ξ=S), and with security constraints only on the message but not on the state (Ξ const). As shown in this example, the additional secrecy constraint on the state is clearly harmful and significantly reduces the set of achievable rates and distortions.*

## 7. ISAC with Detection-Error Exponents

Radar is not only used to estimate parameters such as vehicle velocities, arrival angles, etc., but is also extensively employed to detect obstacles, or more generally, other terminals. In this context, the sensing problem must be framed as a detection or hypothesis testing problem. This framework allows us to incorporate memory into the channel transition law, analogous to slow-fading channels, thereby making the model more reflective of realistic scenarios. This involves considering multiple hypotheses (e.g., the presence or absence of one or more obstacles), and to ensure the model is fully general, we allow the communication channel to depend on the chosen hypothesis. Such a scenario arises, for example, when an obstacle obstructs the line of sight between the Tx and Rx, thereby significantly altering the communication channel characteristics. Of course, the general model also accommodates simpler cases where the communication channel remains independent of the hypothesis.

In this section, the sensing performance is quantified by detection-error probabilities under the different hypotheses. Since these error probabilities can be made to vanish asymptotically with infinite observation lengths, the focus here will be on the exponential decay rate of these error probabilities. Much of the existing literature on the fundamental limits of ISAC with detection-error exponents has focused on mono-static radar, where sensing (detection) is performed at the communication Tx [57,71,72,73,74,114]. A notable exception is [114], where detection is carried out at the communication receiver.

From an information-theoretic perspective, the detection-error ISAC problem is considerably more challenging than the previously presented distortion-based ISAC setup. The primary difficulty stems from the sensing aspect, as the optimal performance of hypothesis testing systems is not well understood, even without the additional communication component present in ISAC systems. In particular, the simple estimation and communication strategies employed in the distortion-ISAC setup are suboptimal. Instead, the Tx can improve its detection performance by generating inputs according to a smart, sequential strategy that adapts based on previously observed outputs. Specifically, in a multi-hypothesis testing problem, the Tx might initially produce inputs based on a particular distribution, and once the observed signals provide sufficient evidence to discriminate one hypothesis with high confidence, it can switch to a different input distribution that better discriminates among the remaining hypotheses.

Adaptive systems are complex to implement, which motivates the practical interest in excluding them from certain considerations. When restricting attention to non-adaptive strategies, similar closed-form expressions for the fundamental ISAC performance limits and trade-offs can be derived, as in the distortion ISAC setup.

The next subsection explains the model both for the adaptive and non-adaptive coding scenarios, and is followed by a section presenting the existing information-theoretic results on ISAC with detection exponents. The last subsection has a slightly different flavor for the sensing task and the goal is to detect a change point.

### 7.1. The Memoryless Block Model

**Figure 19:**
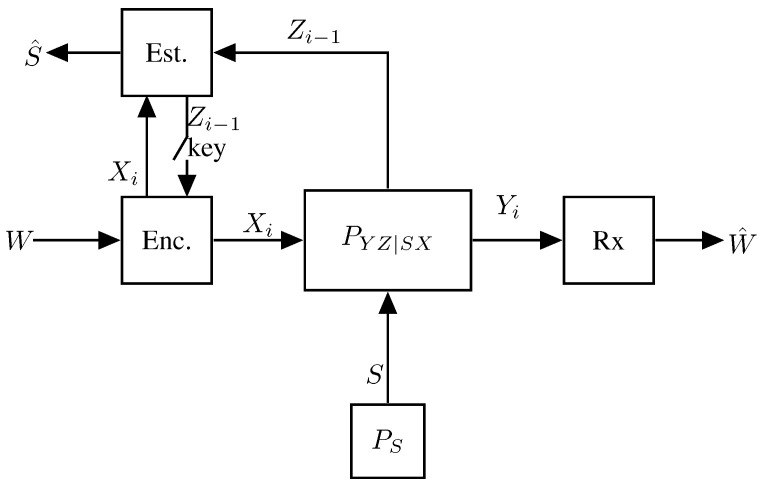
ISAC with a detection sensing problem.

Consider the model in Figure 19, where the Tx wishes to communicate a message to a Rx over a channel that depends on a single parameter θ∈Θ, for Θ a finite set, and at the same time aims to determine this parameter based on the backscattered (generalized-feedback) signals. The parameter is assumed to take value in a discrete and finite set, transforming the sensing problem into a hypothesis testing/detection problem. For a given parameter *θ* in a finite set Θ, the communication channel to the Rx as well as the generalized feedback to the Tx are assumed to be stationary and memoryless, and are described by a joint transition law $P_{YZ|X}^{\theta }$. In the information-theory literature, such a communication channel with a fixed but à prior unknown parameter *θ* is known as a *compound channel* [20,115].

The switch indicates whether the Tx can employ adaptive/closed-loop coding or non-adaptive/open-loop coding. More precisely, if the switch is closed, the *i*-th channel input $X_{i}$ can depend on the previous generalized feedback signals $Z_{1},\ldots ,Z_{i-1}$, and if the switch is open then all inputs only depend on the message *W*.

Formally, the Tx produces the channel inputs either as (in the case of non-adaptive coding)

(74) $$X^{n}=\phi^{\left(n\right)}\left(W\right)$$

or as (in the case of adaptive coding)

(75) $$X_{i}=\phi_{i}\left(W,Z_{1},\ldots ,Z_{i-1}\right),\;i=1,\ldots ,n,$$

where $\phi^{\left(n\right)}$ and $\phi_{1},\ldots ,\phi_{n}$ are encoding functions on appropriate domains. The Tx further guesses the parameter *θ* as

(76) $$\hat{\theta }=h\left(X_{1},\ldots ,X_{n},Z_{1},\ldots ,Z_{n}\right),$$

using some appropriate detection function h(·). As before, the Rx decodes the transmitted message using an appropriate decoding function: $\hat{W}=g\left(Y_{1},\ldots ,Y_{n}\right)$.

Communication performance is measured as before in terms of rate *R* of message *W*, where the Tx and Rx have to be designed in a way that the decoding error probability $\mathrm{Pr}[\hat{W}\neq W|\theta ]$ vanishes asymptotically when the blocklength *n* increases under *any of the hypothesis* θ. In this sense, the communication rate is defined in the same way as for the compound channel [20,115].

Sensing performance is measured in terms of the asymptotic detection-error exponents

(77) $$E_{\theta }≜-\frac{1}{n}\log\mathrm{Pr}\left[h\left(Z^{n},X^{n}\right)\neq \theta |\theta \right],\;\theta \in \Theta ,$$

where the conditioning on *θ* simply indicates that the $Z^{n}$ sequence is generated from $X^{n}$ according to the memoryless law $P_{Z|X}^{\theta }$.

Different requirements on the detection-error exponents have been considered in the literature [57,71,72,73,74]. We summarize the requirements in the following definition.

**Definition** **7.** *Let Θ={0,1}. Then we say that a rate–detection-error exponent (R,D) is achievable* in the Stein setup *if there exists a sequence (in the blocklength n) of encoding, decoding and detection functions so that the following three conditions are satisfied simultaneously:*

(78) $$\lim_{n\to \infty }\mathrm{Pr}\left[\hat{W}\neq W\right]=0$$

(79) $$\lim_{n\to \infty }\mathrm{Pr}\left[h\left(Z^{n},X^{n}\right)\neq 0|\theta =0\right]=0$$

(80) $$E_{1}\geq D.$$

*Similarly, the triple $\left(R,D_{0},D_{1}\right)$ is achievable* in a exponent-region sense, *if above sequences exist so that* (78) *holds, as well as*

(81) $$\begin{array}{ccc} E_{\theta } & \geq & D_{\theta },\;\theta \in \Theta . \end{array}$$

*Let now Θ be arbitrary. Then, the rate–detection-exponent pair (R,D) is called achievable* in the symmetric setup *if encoding, decoding and detection functions exist so that* (78) *holds and*

(82) $$\begin{array}{ccc} \min_{\theta \in \Theta }E_{\theta } & \geq & D. \end{array}$$

*To distinguish between the adaptive and non-adaptive case we will add the superscripts ^*ad*^ and ^*nad*^ to the exponents and write $D^{\mathrm{ad}}$ and $D^{\mathrm{nad}}$.*

In contrast to the model described here, the works in [71,72,73] imposed maximum error probability conditions over the messages both for the decoding error probabilities as well for the detection error probabilities. It turns out that the setup of achievable rate–detection exponent(s) is the same under both average and maximum error probabilities as long as one requires that all error probabilities vanish asymptotically.

### 7.2. Results on the Block Model

We first focus on the model where coding at the Tx is restricted to be non-adaptive. Combining the results in [57,71,72,73,74], we obtain the following theorem:

**Theorem** **14.** *Under non-adaptive coding, we have the following information-theoretic results for the rate–detection-exponent regions in the Stein setup, the symmetric setup, and in the exponents region sense.*

1. *In the* Stein setup, *a non-negative rate–detection-error pair $\left(R,D^{\mathrm{nad}}\right)$ is achievable if, and only if,*
  (83) $$\begin{array}{ccc} R & \leq & \min_{\theta \in \Theta }I_{P_{Y|X}^{\theta }}\left(X;Y\right), \end{array}$$
  (84) $$\begin{array}{ccc} D^{n}ad & \leq & \sum_{x}P_{X}\left(x\right)D\left(P_{Z|X}^{0}\left(\cdot |x\right)∥P_{Z|X}^{1}\left(\cdot \right|x\right) \end{array}$$
2. *In the* exponent-region sense, *a non-negative rate–detection-error pair $\left(R,D^{\mathrm{nad}}\right)$ is achievable if, and only if, for some input distribution $P_{X}$:*
  (85) $$\begin{array}{ccc} R & \leq & \min_{\theta }I_{P_{Y|X}^{\theta }}\left(X;Y\right) \end{array}$$
  (86) $$\begin{array}{ccc} D_{1}^{nad} & \leq & \min_{\begin{array}{c} {\bar{P}}_{Z|X}:\\ E_{P_{X}}\left[D({\bar{P}}_{Z|X}∥P_{Z|X}^{0})\right]\leq D^{n}ad_{0} \end{array}}E_{P_{X}}\left[D\left({\bar{P}}_{Z|X}∥P_{Z|X}^{1}\right)\right] \end{array}$$
3. *In the* symmetric setup, *a non-negative rate–detection-exponent pair $\left(R,D^{\mathrm{nad}}\right)$ is achievable if, and only if, for some input distribution $P_{X}$:*
  (87) $$R\leq \min_{\theta \in \Theta }I_{P_{Y|X}^{\theta }}\left(X;Y\right),$$
  (88) $$D^{\mathrm{nad}}\leq \min_{\theta \in \Theta }\;\min_{a^{\prime }\in \Theta \setminus \theta }\;\max_{l\in [0,1]}-\sum_{x}P_{X}\left(x\right)\log\left(\sum_{z}{\left(P_{Z|XS}^{\theta }\left(z|x\right)\right)}^{l}{\left(P_{Z|XS}^{a}\left(z|x\right)\right)}^{1-l}\right).$$

As already mentioned, exactly characterizing the fundamental limits under adaptive coding seems a very challenging problem and for the moment only achievability results are known [73], which, however, prove the superiority of adaptive coding over non-adaptive coding. Note that it has been known for a long time that for the compound channel, adaptive coding increases communication rate because it allows the Tx to learn the hypothesis with high probability and then adapt the input distribution (and thus the code construction) to the actual transition law of the communication channel. This idea allows the following result to be obtained ([73], Theorem 5).

**Theorem** **15.** *Under adaptive coding, a rate–detection-exponent pair $\left(R,D^{ad}\right)$ is achievable in the symmetric setup if for any θ∈Θ there exists an input distribution $P_{X}$ so that*

(89) $$R\leq I_{P_{Y|X}^{\theta }}\left(X;Y\right),$$

(90) $$D^{\mathrm{ad}}\leq \min_{a\neq \theta }\max_{l\in [0,1]}-\sum_{x}P_{X}\left(x\right)\log\left(\sum_{z}{\left(P_{Z|X}^{\theta }\left(z|x\right)\right)}^{l}{\left(P_{Z|X}^{a}\left(z|x\right)\right)}^{1-l}\right).$$

As mentioned in [73], a further improved region can be achieved by using adaptive strategies also to improve the sensing parts, not only the communication parts. For a more detailed discussion, see [73].

### 7.3. Sequential (Variable-Length) ISAC with Detection-Exponents

In [116], a variable-length version of the ISAC setup with detection-error exponents in the symmetric setup is considered. In this variable-length version, the transmission duration is not fixed from the beginning, but varies as a function of the generalized feedback signals, which in this case, has to coincide with the Rx’s channel outputs to ensure synchronization of the communication. After each time *t*, the Tx will decide based on the past channel outputs $Y_{1},\ldots ,Y_{t}$ whether to stop or continue communication. Let *T* be the random time where transmission stops. The model in [116] imposes that *T* be smaller than a given threshold *n* with high probability.

Given that the communication duration is random, the number of transmitted message bits, and thus the rate of communication, are also allowed to be random. In fact, the message bits are supposed to consist of a stream of i.i.d. Bernoulli-1/2 bits ${\left\{U_{i}\right\}}_{i=1}^{\infty }$, and an increasing sequence of numbers ${\left\{M_{t}\right\}}_{t=1}^{T}$, which indicates at each time *t*, how many information bits have been transmitted until then and have to be decoded at the Rx if transmission stops at time *t*. Since transmission stops at time *T*, the Rx has to decode $W_{T}$ information bits. The rate is defined as

(91) $$R_{T}=\frac{W_{T}}{n},$$

where recall that *T* is the stopping time of the communication and *n* is the given constraint (upper bound) on this stopping time.

Formally, the encoder, decoder, and state detector are described as follows:

- At each time t=1,2,…, the Tx forms the channel input as $X_{t}=f_{t}\left(U_{1},\ldots ,U_{W_{t}},Z^{t-1}\right)$, for an appropriate encoding function $f_{t}$;
- At the end of the transmission, the Tx guesses the state as $\hat{\theta }=h\left(X^{T},Z^{T}\right)$, for an appropriate guessing function *h*;
- At the end of transmission, the Rx decodes the transmitted message bits as ${\hat{U}}^{W_{T}}=g\left(Y_{1},\ldots ,Y_{T}\right)$ for an appropriate decoding function *g*.

**Definition** **8.** *A rate–detection exponent $\left(R,D_{VL}\right)$ is achievable in this* variable-length setup *if there exists a sequence (in the blocklength constraint n) of stopping rules, encoding functions, decoding functions, and state guessing functions, as defined above, such that:*

(92) $$\lim_{n\to \infty }\max_{\theta \in \Theta }\max_{u}\mathrm{Pr}\left[T>n\right]=0$$

(93) $$\lim_{n\to \infty }\min_{\theta \in \Theta }\min_{u}\mathrm{Pr}\left[R^{\left(n\right)}\geq R\right]=1$$

(94) $$\lim_{n\to \infty }\max_{\theta \in \Theta }\max_{u}\mathrm{Pr}\left[{\hat{U}}^{W_{T}}\neq U^{W_{T}}|\theta ,U^{W_{T}}=u\right]=0,$$

(95) $$\underset{n\to \infty }{\underline{\lim}}-\frac{1}{n}\log\max_{\theta \in \Theta }\max_{u}P\left[\hat{\theta }\neq \theta \;|\;\theta ,U^{W_{T}}=u\right]\geq D_{VL}.$$

A set of achievable rate–detection-exponent pairs $\left(R,E_{VL}\right)$ for the described setup was presented in [116]:

**Theorem** **16.** *All rate–detection exponents $\left(R,D_{VL}\right)$ that for each θ∈Θ satisfy the following two conditions for some choice of $P_{X}$ (which can depend on θ)*

(96) $$\begin{array}{cc} R & \leq I(P_{X},P_{Y|X}^{\theta }) \end{array}$$

(97) $$\begin{array}{cc} D_{\mathrm{VL}} & \leq \min_{\theta^{\prime }\neq \theta }E_{P_{X}}\left[D\left(P_{Z|X}^{\theta^{\prime }}∥P_{Z|X}^{\theta }\right)\right] \end{array}$$

*are achievable.*

This result looks similar to the achievability result in Theorem 15, where however the variable-length coding allows an improvement in the detection-error exponent from the Chernoff information in (90) to the Kullback–Leibler divergence in (97). An example in [116] illustrates well this benefit of variable-length coding by means of a numerical plot.

### 7.4. Sequential (Variable-Length) ISAC with Change-Point Detection

A related model has also been considered in [75]. Communication again takes place over a fixed block of *n* channel uses. However, the channel starts in the state θ=0 and at a random time *ν*, it will change to a state θ=1. The goal of the state estimator is to detect this change point *ν* with smallest delay. So, detection is variable-length as in the previous subsection, however data communication is fixed-length over *n* channel uses. Inputs have to be generated in an non-adaptive fashion.

Formally, the Tx generates its inputs as $X^{n}=f^{\left(n\right)}\left(W\right)$, where *W* is a uniform message of rate *R* and *ϕ* an encoding function of appropriate domain. The Rx guesses the message *W* as $\hat{W}=g\left(Y^{n}\right)$ using a guessing function *g*. We again assume perfect feedback Z=Y and the Tx thus estimates the change point using a stopping rule based on all inputs $X^{n}$ and the past outputs $Y_{1},\ldots ,Y_{t}$. We denote the estimate of the change point by the random variable N∈{1,…,n+1}, where n+1 indicates that the channel did not change state. Notice that *N* being a stopping rule based on the inputs and previous outputs, formally means that each event N=i is measurable with respect to $Y^{i}$ and $X^{n}$.

As usual, decoding error probability $\mathrm{Pr}[\hat{W}\neq W]$ is required to tend to 0 as the blocklength n→∞. Typical performance measures for the change-point detection problem are the *false alarm rate* (*FAR*), which in the present setup, should be defined as

(98) $$\mathrm{FAR}=\underset{n\to \infty }{\bar{\lim}}\max_{w}\frac{1}{E_{\infty }\left[N|x^{n}\left(w\right)\right]}.$$

and the *worst-case average detection delay* (*WADD*), which here is defined as:

(99) $$\mathrm{WADD}=\underset{\nu \geq 1}{\sup}\underset{n\to \infty }{\bar{\lim}}\;\max_{w}\;\mathrm{ess}\;\sup_{Y^{\nu -1}}E_{\nu }\left[{\left(N-\nu +1\right)}^{+}|x^{n}\left(w\right),Y^{\nu -1}\right].$$

Here, we defined $E_{\nu }\left[\cdot \right]$ as the expectation operator assuming that the change point is at time *ν*. Similarly, $E_{\infty }\left[\cdot \right]$ denotes the expectation when the state of the channel never changes and remains θ=0 throughout.

**Definition** **9.** *The pair (R,Δ) is called achievable in this quickest change-point detection problem, if for arbitrary small α>0, there exists a sequence (in the blocklength n) of stopping rules N, encoding functions $f^{\left(n\right)}$ and decoding functions g, such that the following conditions are satisfied:*

(100) FAR≤α

(101) $$\mathrm{WADD}\leq \frac{\alpha }{\Delta }$$

(102) $$\lim_{n\to \infty }\mathrm{Pr}\left[\hat{W}\neq W\right]=0.$$

In above definition, Δ describes the ratio between the FAR and the WADD.

The work in [75] establishes a set of achievable (R,Δ) pairs by using sub-block composition codes [117], which not only ensure a given empirical statistics (type) across any given codeword $x^{n}\left(w\right)$ but also within each sub-block.

**Theorem** **17.** *For any choice of $P_{X}$, all pairs of (R,Δ) are achievable that satisfy*

(103) R≤I(X;Y)

(104) $$\Delta \leq E_{P_{X}}\left[D\left(P_{Y|X}^{1}∥P_{Y|X}^{0}\right)\right].$$

## 8. Conclusions and Future Research Direction

In this work, we revisited several models of integrated sensing and communication (ISAC) and information-theoretic results on their fundamental performance limits and the tradeoffs between sensing and communication. These results emphasize the dual role of signals in estimating channel characteristics and enabling communication. We began by analyzing a point-to-point communication setup, where a simple modification of the traditional telecommunication framework allows integrated sensing at the transmitter or receiver. We then reviewed the extended models for network scenarios such as the broadcast channel, multiple-access channels, interference channels, and device-to-device communication. As we have seen, in these network scenarios, the backscattered feedback signals not only enable sensing at the Txs but can also be leveraged for collaborative transmissions of sensing and communication data in future blocks. This improves both the communication and sensing performance metrics and allows new improved tradeoffs and improved overall efficiency. To fully exploit the concept of collaborative communication and sensing in these network ISAC scenarios, advanced coding schemes involving joint source-channel coding schemes are needed. While a large number of results has already been obtained on ISAC systems with distortion constraints, important problems remain open, in particular, single-letter or numerical solutions for channels with memory as well as improved coding schemes and matching converse results for network ISAC systems.

Emerging ISAC systems often face security constraints and the transmitted data or properties of the sensing targets have to be kept secure from external or internal eavesdroppers. Information theorists have studied such ISAC systems with secrecy constraints and determined bounds on the fundamental performance limits, with a focus on secure ISAC coding schemes. We presented these secure ISAC coding schemes and the corresponding securely achievable rate–distortion tuples. Results for different setups were reported: the first setup only requires that part of the data be kept secret, while the second setup imposes the more stringent constraint that in addition to the data, part of the sensing information should also remain unknown to an external eavesdropper. Various future research directions are still open on the information-theoretic framework of secure ISAC, starting with improved achievability and converse results, channels with memory, multi-user systems, and other security constraints regarding the information the eavesdropper obtains about the state sequence.

In addition to the mentioned ISAC scenarios where sensing performance is measured in distortion, this overview article has also considered a fundamentally different ISAC model where the sensing task consists of a detection/hypothesis testing problem. In this setup, the relevant property of the sensing target is characterized by a single finite-valued parameter and the goal of the sensing task is to correctly guess this parameter. The main focus here was on the tradeoff between the achievable data rates and the exponential decay rate of the detection-error exponents. Full characterizations of the set of achievable rate–exponent tuples were obtained under the assumption that the Tx produces its channel inputs in a non-adaptive way. Only preliminary results are available under adaptive coding, thus leaving an interesting field of future research directions. Additional possible directions for future research include also network scenarios or setups with memory.

Furthermore, key research topics include the role of reconfigurable intelligent surfaces (RISs), high-frequency systems (Terahertz and beyond), computational paradigms, and holographic technologies in ISAC systems. Additionally, practical wireless propagation aspects, such as near-field effects, require focused attention. Relevant recent works addressing these challenges include [118,119,120,121,122,123,124].

In conclusion, the convergence of sensing and communication in ISAC systems promises significant advancements in both fields, but it also presents new technical challenges. As highlighted in [39], these challenges span multiple domains and require a comprehensive and interdisciplinary approach. Addressing these challenges will require innovative adaptations of existing models and the development of new schemes that harmonize communication, sensing, and security requirements in increasingly complex environments. One notable direction is the combination of artificial intelligence (AI) and ISAC, which has been extensively discussed in [125,126]. This integration demands not only algorithmic innovation but also a rigorous theoretical foundation. The information-theoretic literature has proved extremely successful in tackling first standard ISAC models, and one can hope that it will also provide a fertile background using these advanced theoretical foundations.

## Figures and Tables

**Table 1:** A Comprehensive Overview of this Survey.

Category	Result Description	Reference(s)
Sensing as Monostatic Radar	Lemma 1: Optimal estimator for P2P and BC	[56]
	Theorem 1: Exact Capacity–Distortion for Memoryless P2P, asymptotic analysis	[56]
	Strong converse Remark 1	[57]
	Log-Loss Distortion Theorem 2	[58]
	Nonasymptotic P2P, Theorem 3	[59]
	Channel with memory, RL approach Theorem 3	[60]
Sensing as Bi-Static Radar (P2P)	C-D with No CSI at Tx Theorem 4	[21]
	C-D with Strictly Causal CSI at Tx Theorem 5	[61]
	C-D Non-Causal CSI, Gaussian Channel at Tx Theorem 6	[62]
Network-ISAC	General BC Outer Theorem 7 and inner Proposition 1 bounds	[63,64]
	Optimal symbolwise estimator	–
	Outerbounds for MAC Theorem 8	[65,66]
	Innerbound MAC Theorem 9	[65,66,67,68]
	Innerbound D2D Theorem 10	[68]
Secrecy-ISAC	Secrecy–Capacity–Distortion Inner Theorem 11 and Outer Theorem 12 Bounds	[69]
	Secrecy of the Message and the State Theorem 13	[70]
ISAC with Detection-Error Exponents	Non-adaptive Rate–Detection-Exponent Theorem 14	[57,71,72,73,74]
	Adaptive Rate–Detection-Exponent Theorem 15	[73]
	Sequential (Variable Length) Rate–Detection-Exponent Theorem 16	[73]
	Sequential (Variable Length) ISAC with Change Point Detection Theorem 17	[75]

**Table 2:** Comparison between Communication and Sensing Systems.

Communication	Sensing
2.4 GHz	24–79 GHz
Data/Source Transmission	Estimation/Detection
Bit/Signal/Frame Error Rate	Minimum Mean Squared Error (MMSE), Cramer–Rao Bound (CRB)
Distortion	Detection/False Alarm Probability
All Propagation Paths	Line of Sight (LoS)

## Data Availability

No new data were created or analyzed in this study.
